# ZnT8 loss-of-function accelerates functional maturation of hESC-derived β cells and resists metabolic stress in diabetes

**DOI:** 10.1038/s41467-022-31829-9

**Published:** 2022-07-16

**Authors:** Qing Ma, Yini Xiao, Wenjun Xu, Menghan Wang, Sheng Li, Zhihao Yang, Minglu Xu, Tengjiao Zhang, Zhen-Ning Zhang, Rui Hu, Qiang Su, Fei Yuan, Tinghui Xiao, Xuan Wang, Qing He, Jiaxu Zhao, Zheng-jun Chen, Zhejin Sheng, Mengyao Chai, Hong Wang, Weiyang Shi, Qiaolin Deng, Xin Cheng, Weida Li

**Affiliations:** 1 Translational Medical Center for Stem Cell Therapy and Institute for Regenerative Medicine, Shanghai East Hospital, Frontier Science Center for Stem Cell Research, School of Life Sciences and Technology, Tongji University, Shanghai, 200092 China; 2 Tsingtao Advanced Research Institute, Tongji University, Qingdao, 266073 China; 3 State Key Laboratory of Cell Biology, CAS Center for Excellence in Molecular Cell Science, Shanghai Institute of Biochemistry and Cell Biology, Chinese Academy of Sciences, University of Chinese Academy of Sciences, 320 Yueyang Road, 200031 Shanghai, China; 4 Department of Physiology and Pharmacology, Karolinska Institute, 17177 Solna, Sweden; 5 Department of Neurosurgery, Huashan Hospital, Institute for Translational Brain Research, State Key Laboratory of Medical Neurobiology, MOE Frontiers Center for Brain Science, Fudan University, Shanghai, 200032 China; 6 School of Life Science and Technology, ShanghaiTech University, 230 Haike Road, 201210 Shanghai, China; 7 Ministry of Education Key Laboratory of Marine Genetics and Breeding, College of Marine Life Sciences, Ocean University of China, Tsingtao, 266003 China

**Keywords:** Cell death, Embryonic stem cells, Diabetes

## Abstract

Human embryonic stem cell-derived β cells (SC-β cells) hold great promise for treatment of diabetes, yet how to achieve functional maturation and protect them against metabolic stresses such as glucotoxicity and lipotoxicity remains elusive. Our single-cell RNA-seq analysis reveals that ZnT8 loss of function (LOF) accelerates the functional maturation of SC-β cells. As a result, ZnT8 LOF improves glucose-stimulated insulin secretion (GSIS) by releasing the negative feedback of zinc inhibition on insulin secretion. Furthermore, we demonstrate that ZnT8 LOF mutations endow SC-β cells with resistance to lipotoxicity/glucotoxicity-triggered cell death by alleviating endoplasmic reticulum (ER) stress through modulation of zinc levels. Importantly, transplantation of SC-β cells with ZnT8 LOF into mice with preexisting diabetes significantly improves glycemia restoration and glucose tolerance. These findings highlight the beneficial effect of ZnT8 LOF on the functional maturation and survival of SC-β cells that are useful as a potential source for cell replacement therapies.

## Abstract (web summary)

Immature function and fragility hinder application of hESC-derived β cells (SC-β cell) for diabetes cell therapy. Here, the authors identify ZnT8 as a gene editing target to enhance the insulin secretion and cell survival under metabolic stress by abolishing zinc transport in SC-β cells.

## Introduction

Pancreatic β-cell loss and dysfunction underlie diabetes mellitus^1,2^. Stem cell-based cell therapy from human pluripotent stem cells (hPSCs) to functional pancreatic β cells (SC-β cells) offers a promising approach for the treatment of diabetes^3–7^. However, the limited functional maturation of these SC-β cells hampers this strategy as a cell replacement therapy for diabetes^8–10^.

The fragility of SC-β cells remains another major challenge for stem cell-based therapy. Although the encapsulation of SC-β cells could prevent immune destruction and oncogenic transformation, this physical barrier is still susceptible to glucotoxicity and lipotoxicity that annihilate the function and viability of β cells. Therefore, it is essential to maintain transplanted SC-β cell’s function and survival against the harmful environment recurrent in diabetic patients to achieve long-term functionality and stability.

ZnT8, which is encoded by *SLC30A8*, is a zinc transporter, that is mainly expressed in pancreatic β cells^11^. ZnT8 regulates the transportation of zinc influx into insulin granules and facilitates insulin hexamer formation^12,13^. Previous mouse studies with global or β cell-specific ZnT8 knockout showed inconsistent phenotypes of insulin secretion^2,14^ from impaired^15,16^ to unaffected^12,17,18^ and improved insulin secretion^19^. In addition, a lack of ZnT8 in mice can protect β cells against hypoxia and cytokine-induced cell death^20^. Interestingly, transgenic mice harboring the rare human *SLC30A8* LOF variant p. Arg138X shows enhanced insulin secretion capacity of pancreatic β cells^21^. In addition, recent concerted studies have revealed that the rare LOF variants of *SLC30A8* protect against diabetes in human subjects^1,13,22^. In 2014, Flannick, J. et al. found 12 rare *SLC30A8* LOF variants in populations that are widely distributed across Asia, America and Europe. These rare LOF variants reduce the risk of diabetes by 65%^1^. Consistently, in 2019, an exome sequencing revealed another 30 *SLC30A8* variants that protected against diabetes from 5 ancestries^22^. Furthermore, in 2019, Dwivedi, O. P. et al.^13^ conducted an oral glucose tolerance test (OGTT) in carriers with the rare LOF variants of *SLC30A8* in comparison with their non-carrier relatives. *SLC30A8* LOF variant carriers exhibited better insulin secretion capacity during the OGTT than their non-carrier relatives. In the same year, it was reported that downregulation of ZnT8 by RNAi in the human insulinoma cell line protects cells from inflammatory stress^23^. Collectively, the above evidence leads us to hypothesize that ZnT8 could be a potential target to improve SC-β cells function for transplantation.

Furthermore, lipotoxicity and glucotoxicity, as metabolic stresses, are detrimental to human pancreatic β cell survival^24,25^. They are also recurrent in diabetic patients as key challenges to cell replacement therapy by jeopardizing the viability of transplanted SC-β cells. Therefore, it would be valuable to determine whether ZnT8 LOF could protect SC-β cells against lipotoxicity and glucotoxicity.

In this work, we introduce the ZnT8 LOF mutation into the genome of hESCs which are differentiated into SC-β cells. The generated SC-β cells with ZnT8 LOF obtain improved insulin secretion capacity, resist glucotoxicity and lipotoxicity, and show significantly improved glycemic control in diabetic mice, offering an advanced strategy for stem cell-based cell replacement therapy for insulin-dependent diabetes.

## Results

### ZnT8 LOF accelerates functional maturation of SC-β cells

**Fig. 1:**
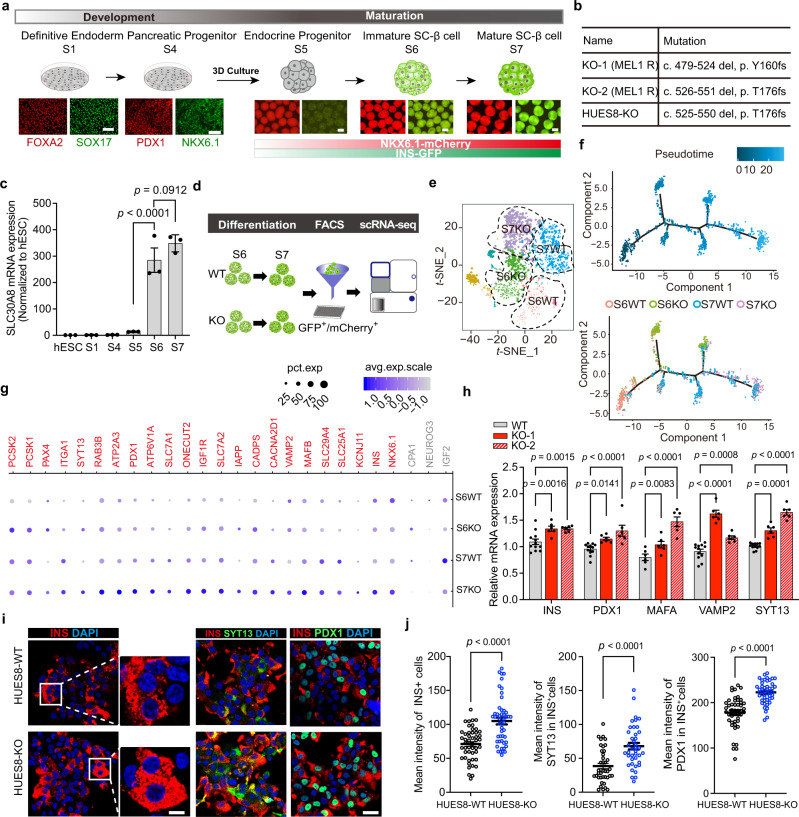
ZnT8 LOF accelerates maturation of SC-β cells. **a** Schematic of pancreatic β-cell differentiation protocol from human embryonic stem cells (hESCs). The key markers of the main stages are illustrated with representative immunofluorescent images. S, stage. Scale bars, 100 μm for S1 and S4; 200 μm for S5, S6, and S7. The red and green bars represent temporal expression of NKX6.1-mCherry and INS-GFP, respectively. **b** Information about three independent *SLC30A8* knockout (KO) hESC lines. **c** Expression profile of *SLC30A8* (normalized to that of hESCs) during the differentiation process. *n* = 3 biological replicates for each stage (one-way ANOVA with Sidak’s test for multiple comparisons). **d** Schematic of the preparation of scRNA-seq by sorting pure WT and KO-1 SC-β cells with the fluorescence of INS-GFP and NKX6.1-mCherry at S6 and S7 respectively. WT, wild type; KO, *SLC30A8* knockout. **e** *t-*SNE projection showing Seurat identified clusters of S6WT (pink), S6KO (green), S7WT (blue), and S7KO (purple). **f** The distribution of pseudotime of S6WT, S6KO, S7WT, and S7KO. **g** Dot plot showing the gene signature changes of S6WT, S6KO, S7WT, and S7KO. **h** qRT-PCR analysis of the expression of maturation related markers in pure SC-β cells (NKX6.1-mCherry^+^/INS-GFP^+^) (WT-MAFA, *n* = 6; WT-INS, PDX1, VAPM2, SYT13, *n* = 12; KO-1, *n* = 6; KO-2, *n* = 6; two-way ANOVA with Dunnett’s test). **i**, **j** Representative immunofluorescent images (**i**) and mean intensity measurements (**j**) for INS, SYT13, and PDX1 of HUES8-WT and HUES8-KO adherent mature SC-β cells (unpaired two-tailed *t* tests; INS, *n* = 49; SYT13, *n* = 40; PDX1, *n* = 47; data points are from five independent experiments for each condition). Scale bar of low magnification, 25 μm; Scale bar of high magnification, 5 μm. Data are presented as mean ± s.e.m. Individual data points are shown for all bar graphs. Source data are provided as a Source Data file.

We first generated a MEL1 NKX6.1:linker2a:mCherry human embryonic stem cell reporter line *(NKX6.1*^*mCherry/mCherry*^*-*INS^GFP^^*/W*^ hESCs) by using a CRISPR/Cas9^26^ knock-in strategy in MEL1 *INS*^*GFP/W*^ hESCs^27^. During stepwise differentiation of hESCs to pancreatic β cells^8,9,28^, the expression of mCherry fluorescence driven by the endogenous *NKX6.1* promoter was observed throughout the pancreatic progenitor cell stage (S4) to mature SC-β cell stage (S7). GFP under the *INS* promoter was initially weakly expressed in the endocrine progenitor cell stage (S5) and then highly expressed in mature SC-β cells (S7) which formed cell cluster structures (Fig. 1a), validating the reporter system.

In light of the protective role of ZnT8 LOF against human diabetes, we investigated whether ZnT8 LOF could improve the function and survival of SC-β cells (Supplementary Fig. 1). First, three independent *SLC30A8* knockout (KO) hESC lines were established by CRISPR/Cas9-based genome editing. The first and second lines were generated by introducing *SLC30A8* nonsense mutations (c. 479–524 del, p. Y160fs) and (c. 526–551 del, p. T176fs) in exon 4 in the aforementioned *NKX6.1*^*mCherry/mCherry*^*-*INS^GFP^^*/W*^ hESC line, and were hereinafter referred to as KO-1 and KO-2 respectively, with the wild type counterpart as WT (Fig. 1b). A third knockout cell line was generated from a different hESC line, HUES8, by introducing the nonsense mutation (c. 525–550 del, p. T176fs) in exon 4 (hereinafter referred to as HUES8-KO and the wild type counterpart as HUES8-WT, Fig. 1b). Each of these three nonsense mutations caused a premature stop codon. We performed qRT-PCR to examine *SLC30A8* expression in the knockout SC-β cells harboring ZnT8 LOF **(**hereinafter referred to as *SLC30A8*^*−/−*^ SC-β cells) including KO-1, KO-2, and HUES8-KO SC-β cells with their WT controls respectively*.* We found that the expression of *SLC30A8* was largely diminished in all three KO SC-β cell lines (Supplementary Fig. 2a). Moreover, immunostaining with a ZnT8 antibody was performed on the WT, KO-1, and KO-2 cells. ZnT8 protein was undetectable in KO-1 and KO-2 cells (Supplementary Fig. 2b). Collectively, these results confirmed that these three lines show complete ZnT8 knockout.

Compared to the WT, ZnT8 LOF affected neither the morphology nor the self-renewal of hESCs. Gene expression of pluripotent stem cell markers and capacity of teratoma formation was comparable between the KO-1 and WT hESCs (Supplementary Fig. 3a, b). Next, we examined whether ZnT8 LOF affected cellular differentiation from definitive endoderm to mature SC-β cells at each stage. ZnT8 LOF had no obvious effect on SC-β cell differentiation efficiency at the definitive endoderm stage (Supplementary Figs. 3c, d and 4a, b), pancreatic progenitor stage (Supplementary Figs. 3e, f and 4c, d) or immature or mature SC-β cell stages (Supplementary Figs. 3g–i and 4e, f). These data, combined with the observation that *SLC30A8* is specifically expressed during the maturation process (from S6 to S7) (Fig. 1c), confirmed that ZnT8 LOF does not affect the cell fate specification of SC-β cells (Supplementary Figs. 3j and 4g).

To investigate the effect of ZnT8 LOF on SC-β cell maturation, we performed single-cell RNA-seq at both S6 (immature β cell) and S7 (mature β cell) for the WT (S6WT, S7WT) and KO-1 (S6KO, S7KO) SC-β cells by sorting out GFP and mCherry double-positive SC-β cells with high *INS* and *NKX6.1* expression (Fig. 1d). All cells were clustered into seven clusters using *t*-distributed stochastic neighbor embedding (*t*-SNE) (Supplementary Fig. 5a). We first excluded 3 small clusters (Cls4, 5 and 6) from downstream analysis based on their low correlation with the rest of the cells indicating the low-quality of these clusters (Supplementary Fig. 5b). The remaining four major clusters, from 0 to 3 mainly comprising S7WT, S6KO, S7KO and S6WT with 283, 331, 424 and 324 cells respectively, showed specific gene expression profiles (Fig. 1e). In order to understand the molecular features of each cluster, we identified the differentially expressed genes (DEGs) for pair-wise comparisons, i.e., S6WT *vs*. S6KO, S7WT *vs*. S7KO, S6WT *vs*. S7WT, and S6KO *vs*. S7KO, resulting in four corresponding groups of genes (i.e., Group 1–4) with gene expression characteristics under biological conditions. Group 1 (S6WT *vs*. S6KO) and Group 2 (S7WT *v*s. S7KO) represented the genes altered between the WT and *SLC30A8*^*−/−*^ SC-β cells at S6 or S7. At S6, the expression of the pancreatic progenitor marker *SOX4* was higher in the WT cells, while the genes such as *PCSK1* and *PCSK2*, which are related to β cell function, were enriched in the S6KO SC-β cells (Supplementary Fig. 5c). At S7, genes important for β cell function and maturation, such as *INS*, *PDX1* and *NKX6.1* were up-regulated in the S7KO SC-β cells (Supplementary Fig. 5c). Group 3 (S6WT *vs*. S7WT) and Group 4 (S6KO *vs*. S7KO) represented the genes altered in the WT or KO SC-β cells during the maturation process. The expression of genes such as *SLC7A2*, *ONECUT2* and *IGF1R* was higher in the KO cells (Supplementary Fig. 5d). Notably, *MAFB*, expressed in mature humans islet β cells, was specifically enriched in S7KO β cells (Supplementary Fig. 5d).

Next, we applied Monocle v2.0 to reconstruct the maturation process to further illustrate the effects of ZnT8 LOF during the increased maturation process in more detail and ordered all cells from 4 major clusters along the pseudotime trajectory. After annotating the cell identity and summarizing the distribution of S6WT, S6KO, S7WT, and S7KO cells along the pseudotime trajectory, we found that both S6KO and S7KO cells were overrepresented in the maturation trajectory compared to the corresponding S6WT and S7WT cells, suggesting that the maturation process is accelerated in the *SLC30A8*^*−/−*^ SC-β cells (Fig. 1f). We then plotted several genes involved in SC-β cell maturation from both the WT and *SLC30A8*^*−/−*^ cells. Interestingly, genes related to β cell function such as *VAMP2*, *PCSK2* and *PCSK1* had significantly increased expression levels and proportions in either S6KO or S7KO or both. Importantly, *ITGA1* (CD49a), a recently identified surface marker for functional and mature β cells derived from hPSCs^29^, was also significantly enriched in the *SLC30A8*^*−/−*^ SC-β cells at both S6 and S7. By contrast, the expression of *CPA1* and *NEUROG3*, which are enriched in pancreatic progenitors, was significantly decreased in the *SLC30A8*^*−/−*^ SC-β cells (Fig. 1g).

To validate the single-cell RNA-seq results, we measured the relevant benchmarks in the SC-β cells derived from all three KO cell lines by qRT-PCR, immunofluorescence assay, and insulin content quantification. First, qRT-PCR was performed to validate the up-regulated function-related genes revealed by scRNA-seq in the SC-β cells from the KO-1 and KO-2 cell lines (Fig. 1h). Second, the insulin content per cell was measured in both the KO-1 and KO-2 SC-β cells and revealed significantly elevated insulin levels per cell in both the KO-1 and KO-2 β cells (Supplementary Fig. 6a). This finding was consistent with the increased insulin transcript levels detected by scRNA-seq. Third, we performed immunofluorescence assays using the antibodies against insulin, SYT13 and PDX1 in the HUES8-KO and KO-1 SC-β cells along with their WT controls, and the quantification of the signal intensity revealed increased expression of all of these markers (Fig. 1i, j and Supplementary Fig. 6b, c). Altogether, these data suggested that ZnT8 LOF accelerates the functional maturation of SC-β cells.

### ZnT8 LOF improves glucose-stimulated insulin secretion

**Fig. 2:**
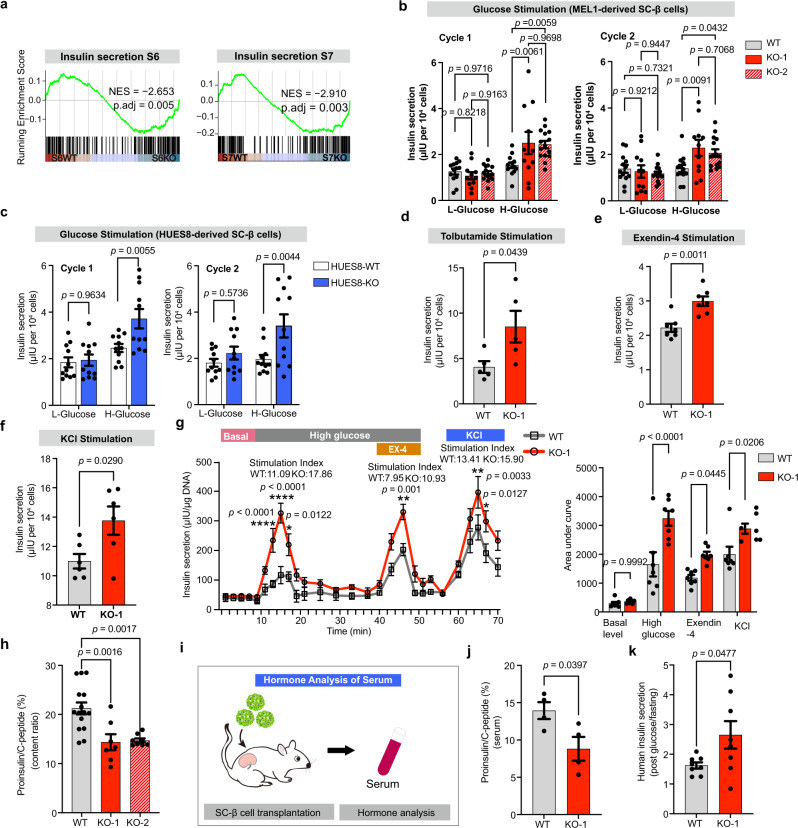
ZnT8 LOF promotes insulin secretion and proinsulin conversion in SC-β cells. **a** Gene set enrichment analysis (GSEA) of insulin secretion genes in S6WT versus S6KO and S7WT versus S7KO cells. **b** Static GSIS (glucose-stimulated insulin secretion) of WT (*n* = 13 for each group), KO-1 (*n* = 11 for each group) and KO-2 (*n* = 15 for each group) SC-β cells (two-way ANOVA with Tukey’s test for multiple comparisons). **c** Static GSIS of HUES8-WT and HUES8-KO SC-β cells (two-way ANOVA with Sidak’s test for multiple comparisons, *n* = 11). **b**, **c** Data are expressed as insulin secretion levels upon high-glucose (20 mM) versus low-glucose (2 mM) stimulation. **d**–**f** Insulin secretion upon tolbutamide (200 μM) (**d** *n* = 5), exendin-4 (**e** *n* = 7) and KCl (**f** *n* = 6) stimulations. **g** Dynamic insulin secretion (perifusion) of WT and KO-1 SC-β cells in response to various secretagogues (glucose, exendin-4, and KCl). Gray line, WT SC-β cells; red line, KO-1 SC-β cells (two-way ANOVA with Sidak’s test for multiple comparisons, *n* = 7). The stimulation index is calculated as the fold change in AUC relative to basal. **h** The ratio of total proinsulin to total C-peptide in WT and KO-1, KO-2 SC-β cells (WT, *n* = 15; KO-1, *n* = 7; KO-2, *n* = 8; One-way ANOVA with Dunnett’s test). **i** Schematic of in vivo function assays of the transplanted SC-β cells. **j** Ratios of human proinsulin to human C-peptide in the serum of the mice transplanted with WT or KO-1 SC-β cells (*n* = 4). **k** The fold change of human insulin secretion upon glucose stimulation relative to that of 16h-fasting (*n* = 8). Unpaired two-tailed *t* tests was used to analyze for **d**–**f**, **j** and **k**. Data are presented as mean ± s.e.m. Individual data points are shown for all bar graphs. Source data are provided as a Source Data file.

Pancreatic β cells gain the capacity of glucose-stimulated insulin secretion (GSIS) as they mature^30^. Single-cell RNA-seq data showed that several insulin secretion pathway-related genes, such as *NKX6.1*, *PDX1*, *IGF1R*, *VAMP2*, *SYT13*, *MAFB*, *CADPS* and *INS* were up-regulated in *SLC30A8*^*−/−*^ SC-β cells. (Fig. 1g). Gene set enrichment analysis (GSEA) also confirmed that the insulin secretion pathway was more enriched in the *SLC30A8*^*−/−*^ SC-β cells than in the WT cells at both S6 and S7 (Fig. 2a). Moreover, pancreatic development, glucose homeostasis, carbohydrate homeostasis, and insulin secretion were among the top 10 enriched biological processes from Gene Ontology (GO) enrichment analysis in S7KO (relative to S7WT) (Supplementary Fig. 6d). Therefore, our single-cell RNA-seq demonstrated that ZnT8 LOF contributes to the upregulation of insulin secretion-related genes and pathways.

To verify whether ZnT8 LOF improves insulin secretion upon glucose stimulation, we performed GSIS assays separately in the KO-1, KO-2, and HUES8-KO cells with their WT controls. Insulin secretion upon glucose stimulation was measured with the GSIS assay by scoring the ratio of insulin release in high glucose (20 mM) to that in low-glucose (2 mM). Compared to their WT counterparts, both the KO-1 and KO-2 SC-β cells showed significantly improved insulin secretion and stimulation index (fold over basal) in two consecutive cycles, namely cycle 1 (insulin secretion upon high glucose: WT, 1.53 ± 0.12; KO-1, 2.50 ± 0.48; KO-2, 2.43 ± 0.16; stimulation index: WT, 1.36 ± 0.15; KO-1, 2.23 ± 0.22; KO-2, 2.19 ± 0.23) and cycle 2 (insulin secretion upon high glucose: WT, 1.39 ± 0.16; KO-1, 2.28 ± 0.35; KO-2, 2.06 ± 0.17; stimulation index: WT, 1.06 ± 0.05; KO-1, 2.34 ± 0.38; KO-2, 1.78 ± 0.10) (Fig. 2b and Supplementary Fig. 6e). Moreover, there was no significant difference of insulin secretion upon high glucose between KO-1 and KO-2 (Fig, 2b; cycle 1, *p* = 0.9698; cycle 2, *p* = 0.7068), suggesting that there was no clonal variation affecting GSIS. Similarly, compared to the HUES8-WT counterparts, the HUES8-KO SC-β cells also showed significantly enhanced GSIS in two consecutive cycles (Fig. 2c and Supplementary Fig. 6f). We also measured insulin secretion upon tolbutamide (K_ATP_ channel blocker), exendin-4 and potassium chloride (KCl) stimulation and found that it was moderately elevated in the KO-1 SC-β cells (Fig. 2d–f). We then performed the perifusion assay to monitor dynamic insulin secretion from SC-β cells as reported recently^31^. The fold change of insulin release in high glucose (AUC-stimulation) to basal insulin (AUC-basal) was scored as the stimulation index to measure dynamic insulin secretion upon stimulation. Each experiment was repeated several times (*n* = 7). The stimulation AUC and indices showed that there was a significant increase in insulin secretion in the KO-1 SC-β cells under various stimulations, including high glucose, Exendin-4, and KCl (Fig. 2g). Taken together, these results confirmed that ZnT8 LOF significantly enhances insulin secretion upon glucose stimulation in SC-β cells.

The expression of *PCSK1*, which encodes prohormone convertase PC1/3 that activates the proinsulin to insulin conversion by proteolysis^32–34^, was increased from our single cell transcriptomic profiling at S6, indicating enhanced conversion efficiency of the *SLC30A8*^*−/−*^ mature SC-β cells (Supplementary Fig. 5c). To validate this finding, we assessed the proinsulin conversion efficiency of the KO-1 and KO-2 SC-β cells by comparing the intracellular total proinsulin to C-peptide ratio, and found that the ratio was significantly decreased in the KO-1 and KO-2 SC-β cells, which demonstrated that the insulin conversion efficiency is higher in the *SLC30A8*^*−/−*^ SC-β cells (Fig. 2h; KO-1, *p* = 0.0016; KO-2, *p* = 0.0017). To further confirm this result in vivo, we transplanted both WT and KO-1 SC-β cells into immune compromised mice (SCID-Beige) (Fig. 2i). The serum levels of human proinsulin and C-peptide were measured. Notably, the human proinsulin/C-peptide ratio was significantly decreased in the mice with *SLC30A8*^*−/−*^ SC-β cell transplants (8.82% ± 1.59%) compared to those with WT SC-β cells (13.94% ± 1.14%), further confirming that proinsulin conversion efficiency was also enhanced in the *SLC30A8*^*−/−*^ SC-β cells (Fig. 2j, *p* = 0.0397) in vivo. Moreover, the in vivo GSIS was significantly increased in the mice transplanted with KO-1 SC-β cells compared to those with WT SC-β cells (Fig. 2k, *p* = 0.0477). Taken together, these results corroborated that *SLC30A8*^*−/−*^ SC-β cells show advanced functional maturation.

### ZnT8 LOF releases zinc inhibition of SC-β cells

**Fig. 3:**
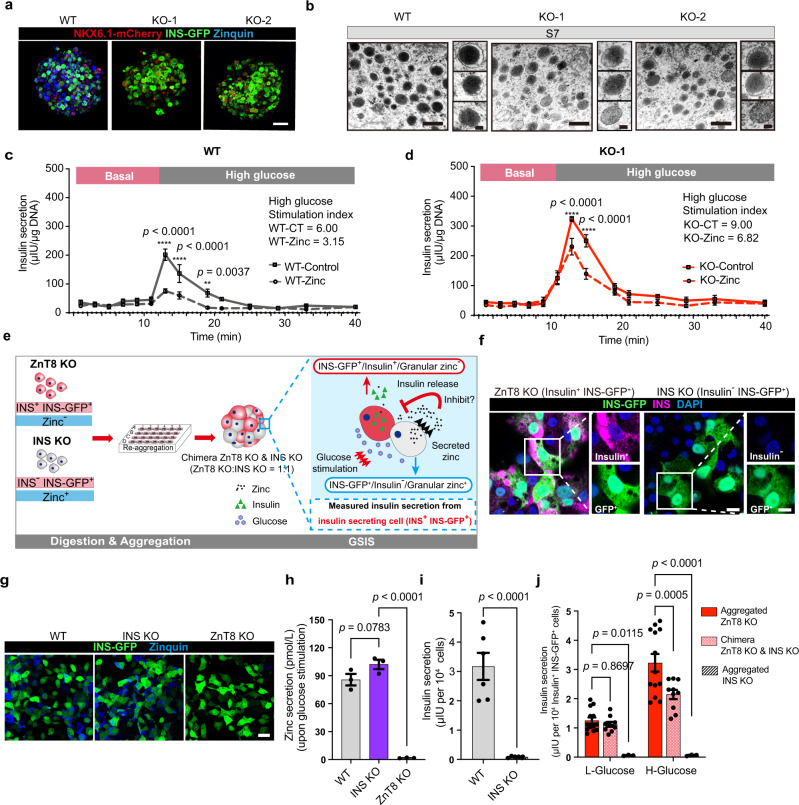
Zinc inhibits insulin secretion upon glucose stimulation. **a** 3D reconstruction of confocal images of Zinquin staining in the WT, KO-1, and KO-2 SC-β cells; scale bar, 25 μm. *n* = 3 independent samples. **b** Transmission electron microscopy images of insulin granules in WT, KO-1, and KO-2 SC-β cells of S7. Scale bars of low magnification, 0.5 μm; Scale bars of high magnification, 0.1 μm. The measurements of mean gray value of insulin granules are in the Supplementary Figure. 7a (*n* = 6). **c**, **d** Dynamic insulin secretion of WT (**c** *n* = 3) and KO-1 (**d** *n* = 3) SC-β cells in the presence of 0 or 100 μM zinc (two-way ANOVA with Sidak’s test for multiple comparisons). **e** Schematic of a chimera model to demonstrate zinc inhibitory effect on insulin secretion. **f** Representative images of INS-GFP and INS in ZnT8 KO and INS KO adherent SC-β cells. Scale bar (low magnification), 10 μm; Scale bar (high magnification), 5 μm. *n* = 3 independent samples. **g** Representative images showing INS-GFP and Zinquin fluorescence of WT, INS KO, and ZnT8 KO adherent SC-β cells. Scale bar, 25 μm. The measurements of zinquin intensity are in the Supplementary Figure. 7d (*n* = 4). **h** Zinc secretion test of WT, INS KO, and ZnT8 KO SC-β cells upon 20 mM glucose stimulation (One-way ANOVA with Dunnett’s test, *n* = 3). **i** Insulin secretion of WT, INS KO SC-β cells upon 20 mM glucose stimulation (unpaired two-tailed *t* tests, *n* = 6). **j** Insulin secretion of Aggregated ZnT8 KO (*n* = 13), Chimera ZnT8 KO & INS KO (*n* = 10) and aggregated INS KO (*n* = 3) SC-β cells in response to Glucose (two-way ANOVA with Sidak’s test). Data are presented as mean ± s.e.m. Individual data points are shown for all bar graphs. Source data are provided as a Source Data file.

To decipher how ZnT8 LOF improves SC-β cell function, we focused on its role in the regulation of zinc transportation. We used a sensitive zinc fluorescence indicator, namely Zinquin, to monitor zinc levels inside the insulin secretory granules^11^. We observed that the typical “dotted” pattern of granular zinc found in the WT SC-β cells was largely missing in the *SLC30A8*^*−/−*^ SC-β cells (Fig. 3a), which led to our hypothesis that the lack of granular zinc might contribute to the enhancement of insulin secretion., Moreover, we measured the mean gray value of the dense core granules for both KO-1 and KO-2 along with the WT control in transmission electron microscopy images. The results showed that the intensity was significantly reduced in both the KO-1 and KO-2 SC-β cells (Fig. 3b and Supplementary Fig. 7a). Similarly, the immature insulin granules in the *SLC30A8*^*−/−*^ SC-β cells appeared paler than those in the WT cells at S6 (Supplementary Fig. 7b, c).

It has been recognized previously that zinc is co-secreted with insulin upon glucose stimulation, and that free zinc plays an inhibitory role in insulin secretion upon glucose stimulation in a pancreatic β-cell line^35^, from primary mouse islets^36^, and from rat β-cells and primary islets^37,38^. To determine whether there is an inhibitory effect of zinc on insulin secretion stimulated by glucose in SC-β cells, we performed perifusion assays to monitor the dynamic insulin secretion upon glucose stimulation in the absence or presence of free zinc, and the assays were repeated several times with the WT and KO-1 SC-β cells. The stimulation index was scored by the fold change of insulin released at high glucose (AUC-high) to basal insulin (AUC-basal) as reported^31^. In WT SC-β cells, zinc treatment conferred a strong inhibitory effect on dynamic insulin secretion upon glucose stimulation (Fig. 3c, WT control = 6.00; WT zinc = 3.15). In the KO SC-β cells, zinc treatment also conferred an inhibitory effect on insulin secretion upon glucose stimulation (Fig. 3d, KO control = 9.00; KO zinc = 6.82). The results demonstrated that zinc has an inhibitory effect on insulin secretion upon glucose stimulation in both the WT and KO SC-β cells. Moreover, the zinc inhibitory effects between the WT and KO SC-β cells were compared. The stimulation index of the WT SC-β cells was decreased by 47.5% by zinc treatment, whereas the stimulation index of the KO SC-β cells was decreased by 24.2% by zinc treatment. Since the overall level of zinc outside the cells is determined by the zinc secreted by the cells and the zinc administered, ZnT8 LOF is expected to decrease total zinc by reducing zinc secretion, which therefore explains why a less prominent inhibitory effect of zinc on KO SC-β cells was observed.

To further confirm the inhibitory effect of secreted zinc from SC-β cells, we designed a chimera model in which secreted zinc was released from INS KO SC-β cells with granular zinc but without insulin, and insulin was released from ZnT8 KO SC-β cells but without granular zinc (Fig. 3e). First, an INS KO MEL1 reporter line (*INS-GFP*^*/−*^ hESCs) was constructed and differentiated into SC-β cells. The INS KO SC-β cells were labeled with INS-GFP and Zinquin staining despite no insulin content (INS-GFP^+^/insulin^−^/granular zinc^+^) (Fig. 3f, g and Supplementary Fig. 7d). Importantly, upon glucose stimulation, the INS KO SC-β cells had the same capacity of zinc secretion as the WT SC-β cells but without insulin secretion (Fig. 3h, i). In contrast, the ZnT8 KO SC-β cells labeled with INS-GFP showed insulin secretion but no granular zinc (INS-GFP^+^/insulin^+^/granular zinc^−^) (Fig. 3f, g and Supplementary Fig. 7d). Upon glucose stimulation, the ZnT8 KO SC-β cells had the capacity of insulin secretion but without significant granular zinc release (Fig.3h). The INS KO SC-β cells and the ZnT8 KO SC-β cells were both dissociated into single cells, mixed at a 1:1 ratio, and then aggregated with AggreWell plates, named as “Chimera ZnT8 KO & INS KO”. Similarly, single ZnT8 KO SC-β cells or INS KO SC-β cells were also aggregated into spheroids as control, and named “Aggregated ZnT8 KO” or “Aggregated INS KO”, respectively. The GSIS assay was performed to test whether secreted zinc has an inhibitory effect on insulin release or not. As a result, upon glucose stimulation with secreted zinc from the INS KO SC-β cells, insulin secretion from the ZnT8 KO SC-β cells in the chimera was significantly decreased (Fig. 3j and Supplementary Fig. 7e). Altogether, our results demonstrated that secreted zinc inhibits insulin secretion upon glucose stimulation.

To determine how the granular zinc level correlated with insulin release, we analyzed insulin levels and granular zinc intensity by visualizing INS-GFP and Zinquin staining in both WT SC-β cell clusters and dissociated SC-β cells (Supplementary Fig. 7f). A significant negative correlation revealed a higher granular zinc content in the weakly INS-GFP-expressing SC-β cells (33.33% ± 2.40%) than in the strongly INS-GFP-expressing SC-β cells (17.33% ± 1.76%) (Supplementary Fig. 7g), indicating that zinc concentration regulates insulin levels in SC-β cells. To further confirm that increased granular zinc suppresses insulin expression, we restored the granular zinc level by ectopically expressing *SLC30A8* via adenovirus transduction in the WT SC-β cells (Supplementary Fig. 7h). The results showed that increased granular zinc indeed suppressed insulin expression levels, as evidenced by the attenuated INS-GFP level (Supplementary Fig. 7i). In contrast, the WT SC-β cells infected with adenovirus carrying mCherry as a control showed no decrease in GFP levels (Supplementary Fig. 7i). These results supported our hypothesis that ZnT8 LOF reduces granular zinc storage, consequently releases the negative feedback inhibition on insulin secretion, and up-regulates insulin expression in SC-β cells.

### ZnT8 LOF SC-β cells resist lipotoxicity and glucotoxicity

**Fig. 4:**
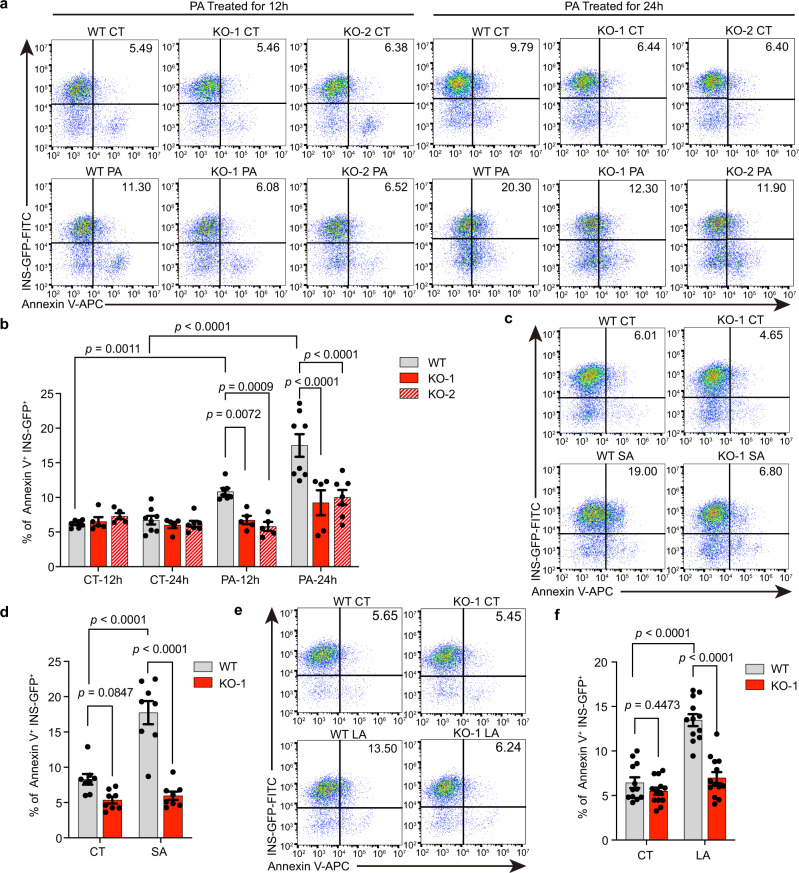
ZnT8 LOF SC-β cells are resistant to lipotoxicity-triggered cell death. **a**, **b** Representative FACS plots (**a**) and quantifications (**b**) of Annexin V^+^/INS-GFP^+^ populations in SC-β cells treated with PA (palmitic acid) or solvent (CT, control) for 12 h (WT, *n* = 7; KO-1, *n* = 5; KO-2, *n* = 5) or 24 h (WT, *n* = 8; KO-1, *n* = 5; KO-2, *n* = 7). **c**, **d** Representative FACS plots (**c**) and quantifications (**d**) of Annexin V staining in WT and KO-1 SC-β cells upon SA (stearic acid) or solvent treatment (*n* = 8). **e**, **f** Representative FACS plots (**e**) and quantifications (**f**) of Annexin V staining in WT (*n* = 12) and KO-1 (*n* = 13) SC-β cells under LA (linoleic acid) or solvent treatment. Two-way ANOVA with Sidak’s test was used in **b**, **d** and **f** for multiple comparisons. Data are presented as mean ± s.e.m. Individual data points are shown for all bar graphs. Source data are provided as a Source Data file.

Lipotoxicity and glucotoxicity are two crucial risk factors associated with β cell failure. Since ZnT8 LOF protects humans against diabetes, we wondered whether ZnT8 LOF wards SC-β cells off lipotoxicity or glucotoxicity. To sufficiently simulate in vivo lipotoxicity in the three *SLC30A8*^−/−^ SC-β cells (KO-1, KO-2, and HUES8-KO) and their corresponding WT controls*,* we applied major representative free fatty acids (FFAs) in humans and mice, including the saturated fatty acids C16:0 (palmitic acid, PA) and, C18:0 (stearic acid, SA), and the unsaturated fatty acid C18:2 (linoleic acid, LA) along with solvent controls^39^. Concentrations used for PA (1.5 mM), SA (600 μM), and LA (150 μM) were used according to previous studies on testing lipotoxicity with these fatty acids in SC-β cells^6,40^ or rodent β cells^41,42^. To avoid the mask of lipotoxicity of long-chain fatty acids by bovine serum albumin in SC-β cell culture medium, we used an albumin-free medium before FFAs treatment^43,44^. Notably, in the KO-1, KO-2 SC-β cells and their WT counterparts derived from the reporter line, the death of SC-β cells was measured by flow cytometry with Annexin V^+^/INS-GFP^+^ double labeling^6,40^. We indeed observed significantly increased cell death triggered by PA, SA, and LA with 12 or 24 h of treatment in the WT SC-β cells compared to solvent control-treated SC-β cells (Fig. 4a–f). Interestingly, the *SLC30A8*^*−/−*^ SC-β cells derived from MEL1 hESCs harboring ZnT8 LOF were resistant to PA (Fig. 4a, b), SA (Fig. 4c, d), and LA (Fig. 4e, f) induced cell death. We also measured necrosis and apoptosis induced by PA, SA, and LA in the HUES8-KO and HUES8-WT SC-β cells respectively, by double-labeling the cells with PI (propidium iodide) and Annexin V. The results demonstrated that ZnT8 LOF significantly decreased the proportions of cells that were undergoing apoptosis (Annexin V^+^) and necrosis (PI^+^/Annexin V^+^) under lipotoxicity induced by PA (Supplementary Fig. 8a, b), SA (Supplementary Fig. 8c, d), and LA (Supplementary Fig. 8e, f). The results demonstrated that ZnT8 LOF protects SC-β cells at multiple time points following the onset of lipotoxicity.

To evaluate glucotoxicity-induced cell death in the WT and KO-1 SC-β cells, we applied a high dose of glucose (35 mM) to the SC-β cell cultures for 24 h to induce glucotoxicity. Cells were analyzed by flow cytometry for Annexin V staining and INS-GFP. The percentage of Annexin V^+^/INS-GFP^+^ cells in the glucose-treated WT SC-β cells was significantly higher than that in the glucose-treated KO-1 SC-β cells (Supplementary Fig. 9a, b), which demonstrated that ZnT8 LOF could also protect SC-β cells from glucotoxicity-induced cell death. The Fragility of SC-β cells in a hostile environment in diabetic patients, such as glucotoxicity and lipotoxicity, is a bottleneck for the efficacy of cell therapy with SC β-cells^45–47^. To address this issue, we simulated pancreatic β cell death caused by glucotoxicity and lipotoxicity (PA, SA, LA) in vitro and our results portrayed that ZnT8 KO SC-β cells may be more resistant to hostile environments with glucotoxicity and lipotoxicity, as observed in diabetic patients with metabolic disorders.

### ZnT8 LOF attenuates ER stress in SC-β cells

**Fig. 5:**
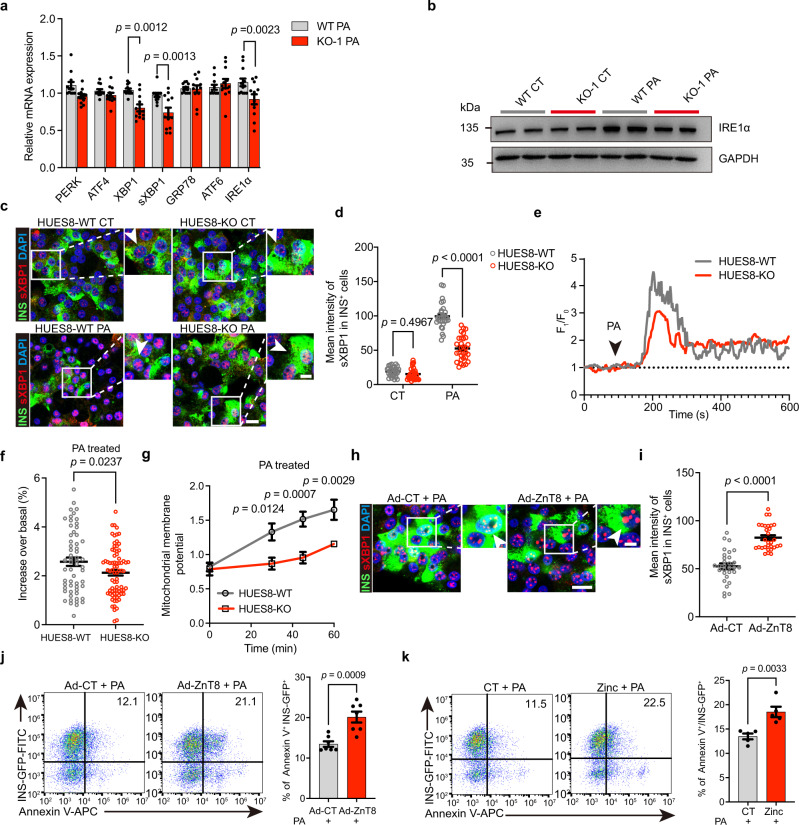
ZnT8 LOF enhances SC-β cell survival by alleviating ER stress. **a** qRT-PCR analysis of the expression of ER stress-related genes for WT and KO-1 SC-β cells in the presence of PA (*n* = 12). **b** Protein expression of WT and KO-1 SC-β cells treated with PA was determined by western blot analysis (*n* = 3 independent experiments). **c**, **d** Representative immunofluorescent images (**c**) and mean intensity measurement (**d**) of sXBP1 in the INS^+^ cells with PA or solvent control treatment (*n* = 30 from three independent experiments). **e** Representative trace of Fluo-4 relative fluorescence intensity (F_1_/F_0_) versus time in a single SC-β cell upon PA stimulation. **f** Maximal peak increase of relative fluorescence intensity upon PA stimulation over an average of baseline fluorescence intensity (0-90 s) (HUES8-WT, *n* = 58; HUES8-KO, *n* = 77 from four independent experiments). **g** Mitochondrial membrane potential measured by JC-10 (mean fluorescence of monomer/aggregate). The samples were treated with PA for 0 (*n* = 4), 30 min (*n* = 6), 45 min (*n* = 7), and 60 min (*n* = 7). **h**, **i** Representative images (**h**) and mean intensity measurement (**i**) of sXBP1 in the INS^+^ cells of HUES8-WT infected with Ad-CT (adenovirus-control) or Ad-ZnT8 (adenovirus-ZnT8) in the presence of PA (*n* = 30 cells from three independent experiments for each condition). **j** Representative FACS plots and quantifications of the percentages of Annexin V^+^/INS-GFP^+^ cells in the WT SC-β cells treated with PA and infected with adenovirus-ZnT8 or adenovirus-control (*n* = 7). **k** Representative FACS plots and quantifications of the Annexin V^+^/INS-GFP^+^ percentages in the SC-β cells in the absence or the presence of ZnSO_4_ (100 μM) (*n* = 5). Two-way ANOVA with Sidak’s multiple-comparisons was used to analyze for **a**, **d** and **g**. Unpaired two-tailed *t* tests were used to analyze for **f**, **i**, **j** and **k**. Scale bars of **c** and **h** are 10 μm (low magnification) and 5 μm (high magnification). Data are presented as mean ± s.e.m. Individual data points are shown for all bar graphs. Source data are provided as a Source Data file.

Lipotoxicity and glucotoxicity trigger ER stress in islet β cells, and ER stress-induced calcium release from the ER lumen to the cytosol results in mitochondrial depolarization which contributes to consequent cell death^48–51^. Since we observed that ZnT8 LOF protects SC-β cells against lipotoxicity and glucotoxicity, we then set out to determine whether ZnT8 LOF could attenuate ER stress in SC-β cells. qRT-PCR was performed to profile the expression of ER stress-associated genes in the WT and KO-1 SC-β cells under metabolic stress. In the presence of PA administration without albumin in the medium, the ER stress-related markers, IRE1α, XBP1, and sXBP1 were dramatically up-regulated in the WT SC-β cells compared to those in the absence of PA (Supplementary Fig. 10a), demonstrating a detrimental effect from lipotoxicity. Moreover, their expression was significantly down-regulated in the ZnT8 KO SC-β cells compared to the WT cells treated with PA in an albumin-free medium (Fig. 5a). As ER stress elevates, sXBP1 is cleaved from XBP1 by IRE1α, and then, the sXBP1 protein translocates to the nucleus^52^. Accordingly, we found that the protein level of IRE1α was significantly down-regulated in the KO-1 SC-β cells compared to the WT cells with PA treatment by western blot (Fig. 5b). Moreover, we detected the level of nuclear entry of sXBP1 by immunostaining, showing that nuclear sXBP1 was significantly decreased in the HUES8-KO SC-β cells compared with the WT cells treated with three types of free fatty acids (FFAs) respectively (Fig. 5c, d and Supplementary Fig. 10b, c), suggesting that ZnT8 LOF ameliorates ER stress induced by lipotoxicity. It has been reported that lipotoxicity-induced ER stress is accompanied by alterations in cytosolic calcium homeostasis^53,54^. Therefore, we measured the dynamic cytosolic calcium level with Fluo-4 relative fluorescence intensity (F_1_/F_0_)^55–57^. After labeling with Fluo-4 AM, consecutive images of the treated cells were taken every 2 s by confocal microscopy for 600 s. The baseline of intracellular calcium level was monitored for 90 s, followed by FFAs treatment. For comparison, a typical trace of a single SC-β cell for HUES8-WT and HUES8-KO obtained with FFAs stimulation was plotted (Fig. 5e and Supplementary Fig. 10d, f). Upon FFAs treatment, the maximal peak increase in the intracellular calcium level was significantly lower for the HUES8-KO SC-β cells than for the HUES8-WT cells (Fig. 5f and Supplementary Fig.10e, g), which indicated that ZnT8 LOF attenuates the increase in cytosolic calcium levels upon lipotoxicity. The resultant mitochondrial membrane potential was measured by JC-10 fluorescence dye. In the HUES8-WT SC-β cells, we observed a sharp increase in the green monomeric to red aggregated fluorescence ratio following short-term exposure to FFAs, which manifested more severe mitochondrial depolarization than that in the HUES8-KO SC-β cells (Fig. 5g and Supplementary Fig. 10h, i). The results were in line with the above results of significantly decreased cell death of the ZnT8 LOF SC-β cells since mitochondrial depolarization is an indicator of cell death. Altogether, these results suggested that ZnT8 LOF attenuates ER stress triggered by lipotoxicity in SC-β cells.

Compared to that of other cell types (2–10 μM), the zinc level in insulin granules of islet β cells (10–20 mM) is highly concentrated^8^. Co-secreted zinc from insulin granules was reported to be a detrimental paracrine effector contributing to consequent cell death^58,59^. Since ZnT8 LOF dramatically reduces granular zinc in SC-β cells, we hypothesized that ZnT8 LOF alleviates ER stress-triggered cell death by releasing zinc detrimental effect. To test this hypothesis, we first enhanced the granular zinc level by overexpressing ZnT8 in SC-β cells under PA treatment, which led to elevated ER stress (Fig. 5h, i and Supplementary Fig.11a) and consequent cell death in these cells (Fig. 5j). Then extra zinc was added to SC-β cells, which elicited ER stress in SC-β cells and exacerbated cell death induced by lipotoxicity (Fig. 5k and Supplementary Fig.11b). Collectively, these results suggested that ZnT8 LOF ameliorates ER stress in SC-β cells by modulating zinc levels.

### ZnT8 LOF SC-β cells improve glycemia restoration in vivo

**Fig. 6:**
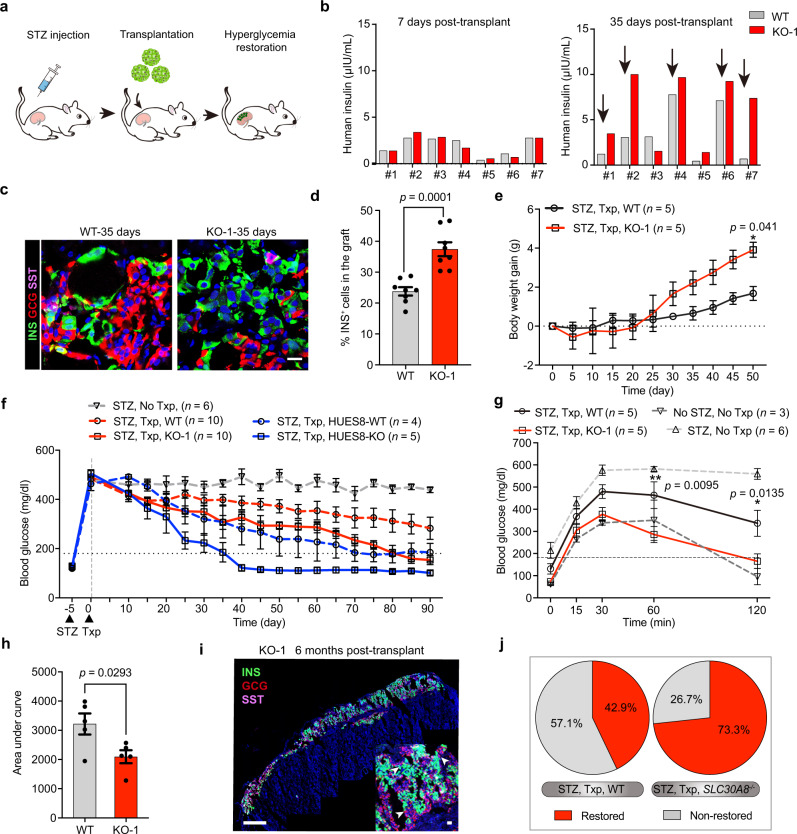
ZnT8 LOF SC-β cells improve glycemic restoration in vivo. **a** Schematic for transplantation of SC-β cells under the kidney capsule of streptozotocin (STZ)- induced diabetic mice. **b** Serum levels of human insulin at day 7 and day 35 post-transplant in the paired animals transplanted with WT or KO-1 SC-β cells (3×10^6^/animal, *n* = 2 per cohort). **c**, **d** Immunostaining (**c**) and INS^+^ cell quantifications (**d**) of WT and KO-1 SC-β cells’ grafts retrieved at day 35 post-transplant; scale bar, 25 μm. *n* = 8. **e** Body weight gain of the diabetic mice transplanted with WT and KO-1 SC-β cells (*n* = 5). **f** Blood glucose levels in randomly fed STZ-induced diabetic mice transplanted with (red dotted line: STZ, WT Txp, *n* = 10; red line: STZ, KO-1 Txp, *n* = 10; blue dotted line: STZ, HUES8-WT Txp, *n* = 4; blue line: STZ, HUES8-KO Txp, *n* = 5) or without (gray dotted line: STZ, No Txp, *n* = 6) SC-β cells. **g**, **h** Intraperitoneal glucose tolerance test (**g**) and AUC analysis (**h**) of the diabetic mice transplanted with (black line: STZ, WT Txp, *n* = 5; red line: STZ, KO-1 Txp, *n* = 5) or without (light gray dotted line: STZ, No Txp, *n* = 6; dark gray dotted line: No STZ, No Txp, *n* = 3) SC-β cells. **i** Representative immunofluorescent images of KO-1 SC-β cell grafts retrieved at 6 months post-transplant. Scale bars are 500 μm (low magnification) and 20 μm (high magnification). *n* = 3 independent samples. **j** Pancake plot illustrating the percentages of hyperglycemia restored (red, blood glucose <180 mg/dl) and unrestored (gray, blood glucose >180 mg/dl) diabetic mice transplanted with SC-β cells at 90 days post-transplant. Two-way ANOVA with Sidak’s test for multiple comparisons for **e** and **g**, unpaired two-tailed *t* tests for **d** and **h**. Data are presented as mean ± s.e.m. Individual data points are shown for all bar graphs. Source data are provided as a Source Data file.

The primary goal of SC-β cells is to cure insulin-dependent diabetes mellitus (IDDM)^46^. To test the in vivo efficacy of *SLC30A8*^*−/−*^ SC-β cells, we induced IDDM in SCID-Beige mice by streptozotocin (STZ) administration. WT or KO-1 SC-β cell clusters containing 3 million cells were transplanted into the kidney capsules of the STZ-induced diabetic mice with hyperglycemia (blood glucose level >400 mg/dl) (Fig. 6a). Human insulin levels in the transplanted mice serum were measured on day 7 and day 35 after transplantation. On day 7, no obvious difference in serum human insulin levels was observed in each independent group (numbered 1–7) of the mice transplanted with WT or KO-1 SC-β cells. On day 35, except in Groups 3 and 5, all the other groups showed a significantly increased serum insulin level in the animals transplanted with KO-1 SC-β cells as compared to the animals transplanted with WT SC-β cells (Fig. 6b). Importantly, immunostaining of grafts on day 35 showed more insulin-positive cells in the KO-1 SC-β cells transplanted mice (Fig. 6c, d). The body weight gain and blood glucose levels were measured every 5 days after transplantation. The mice transplanted with KO-1 SC-β cells showed significantly increased body weight gain and decreased blood glucose levels compared to those transplanted with WT SC-β cells, suggesting improved glycemia restoring the capacity of the KO-1 SC-β cells (Fig. 6e, f). For the intraperitoneal glucose tolerance test (i.p.GTT), mice were fasted for 16 h and consequently challenged with 3 g/kg glucose by intraperitoneal injection. Blood glucose change during the 120 min after glucose intraperitoneal injection revealed that the animals with KO-1 SC-β cells exhibited significantly better glucose tolerance than those with WT SC-β cells, responding to the glucose challenge similar to the nondiabetic untreated mice (Fig. 6g, h).

The kidney grafts of KO-1 SC-β cells were analyzed for immunostaining 6 months after transplantation, which showed an architecture of intermingled insulin^+^ and glucagon^+^ cells and somatostatin^+^ cells (white arrows on the image) similar to human islets (Fig. 6i), demonstrating the long-term stability of SC-β cells with ZnT8 LOF in diabetic mice. To rule out the possibility of β cell retention in the mouse pancreas which might complicate the readout, we assessed by sectioning the pancreas following STZ injury at day 90 post-transplantation. We observed that the islet remnants in the pancreas were mainly composed of glucagon^+^ alpha cells with an absence of the majority of β cells (Supplementary Fig. 12a). Furthermore, nephrectomy was performed to remove the transplanted hESC-derived SC-β cells, after which a drastic elevation in glucose levels was observed (Supplementary Fig. 12b), clearly demonstrating that glucose levels in the transplanted animals were mainly controlled by SC-β cells.

In parallel, to further confirm the improved in vivo efficacy of SC-β cells with ZnT8 LOF from different hESC lines, we transplanted HUES8-WT and KO SC-β cells (each for 3 million) into mice with pre-existing diabetes induced by STZ. Consistent with what was observed with the KO-1 SC-β cells, the HUES8-KO SC-β cells also showed better glycemic control and body weight gain than the HUES8-WT SC-β cells (Fig. 6f and Supplementary Fig. 12c). In total, the AUC of the glycemia controlled by *SLC30A8*^*−/−*^ SC-β cells were significantly lower than the AUC of glycemia controlled by their wild type counterparts (Supplementary Fig. 12d). After transplantation for 90 days, the blood glucose level in 11 of 15 (73.3%) mice transplanted with *SLC30A8*^*−/−*^ SC-β cells was restored to normal (a level below 180 mg/dl), whereas only 6 of 14 (42.9%) mice transplanted with WT SC-β cells showed restored normal glucose levels (Fig. 6j). Notably, the WT and KO-1 SC-β cells are with the knock-in of INS^GFP^^*/W*^ from MEL1(MEL1 *INS*^*GFP/W*^), which is widely used in studies on SC-β cells from hESCs^3,40,60^. It has been reported that the reporter line MEL1 *INS*^*GFP/W*^ is a heterozygous INS hESC line, in which one insulin allele is inserted by *GFP* to label β cell^61^, leading to haploinsufficiency of insulin expression^45^. However, the subsequent effect is the decreased insulin secretion compared to the homozygous SC-β cell^45^, which could explain the better therapeutic effect of HUES8 derived SC-β cells. Altogether, our results provided the evidence that introducing ZnT8 LOF into SC-β cells may represent a promising strategy for preparing source material for cell replacement therapy of diabetes.

## Discussion

Recent breakthroughs in clinical trials have shown encouraging therapeutic results of SC-β cells for the treatment of diabetes^62–64^. Notably, one T1D patient receiving cell therapy with SC-β cells developed by Vertex (VX-880) demonstrated robust insulin secretion from the transplant, improved glycemic control and decreased exogenous daily insulin use (up to a 91% decrease on Day 90 post-transplantation)^62^. The most appealing advantage of SC-β cells lies in the unlimited supply, circumventing the donor scarcity of cadaveric islets for cell therapy^45^. Nevertheless, two common bottlenecks hamper the widespread application of SC-β cells: immature function and fragility to a hostile environment^46,47^. Gene editing has been proposed to not only enhance engraftment, function, and survival, but also enable SC-β cells to evade immune attack^47^. Currently, improvement of SC-β cells by gene editing is also under exploration by the key industrial players, Vertex and ViaCyte^47,65^. However, no progress has been reported on this topic. In this study, we demonstrate that the introduction of ZnT8 LOF in SC-β cells accelerates functional maturation and resists metabolic stress, providing a proof of concept to improve SC-β cells via gene editing.

Previous studies revealed numerous druggable targets for diabetes, such as DPP4 and SGLT2, whose mutations are associated with a decreased risk of diabetes^22^. Inhibitors targeting DPP4 and SGLT2 have been developed as antidiabetic drugs^66,67^. Human rare LOF mutations of ZnT8 decrease the risk of diabetes and improve insulin secretion from pancreatic β cells^1,13,21,22^. Moreover, it is also reported that downregulation of ZnT8 protects pancreatic β cells from cell death induced by inflammatory stress, cytokines and hypoxia^23,68^. Consistently, our study also demonstrates that ZnT8 LOF promotes SC-β cell function and reduces cell death triggered by metabolic stress-induced ER stress. Collectively, our results and previous studies identify ZnT8 as a potential druggable target to improve pancreatic β cell function. Thus, it might be important in future studies to identify ZnT8 inhibitors, which could be utilized as innovative therapeutics for diabetic prevention and treatment. Interestingly, in multiple species, such as Guinea pig *(Cavia porcellus)*, chinchilla *(Chinchilla lanigera)*, naked mole-rat *(Heterocephalus glaber)*, sheep *(Ovis aries)* and cow *(Bos taurus)*, SL*C30A8* has been reported as an inactivated pseudogene, resulting in significantly low islet zinc content^69–73^, implying that this gene is not required for survival. Thus, it will be important to explore the significance of the existence of *SLC30A8* from an evolutionary perspective across different species in future studies.

The ER and mitochondria are two pivotal organelles for the function and survival of β cells^52,74,75^. The ER is the organelle where proinsulin is processed and folded^76^. Under physiological conditions, proteins are properly folded in the ER and misfolded proteins are degraded through endoplasmic reticulum-associated protein degradation (ERAD)^77^. ER stress is up-regulated when the accumulation of misfolded proteins exceeds the capacity of the ER under pathological conditions^78^. More than 50% of the total mRNA of β cells is dedicated to insulin synthesis, rendering it susceptible to ER stress^52,79^. In addition, the ER is essential to maintain calcium homeostasis, disturbances of which may cause metabolic diseases^75,80^. ER stress elevates cytosolic calcium, leading to mitochondrial depolarization and irreversible cell death^81,82^. Excessive free fatty acids (lipotoxicity) or chronic high glucose (glucotoxicity) results in mitochondrial fission and consequently causes β cell death^83^. In addition, mitochondria are mainly involved in GSIS, which is strongly correlated with β-cell functional maturation^60,83–85^.

Notably, β cells have the most abundant zinc level in insulin secretory granules compared to other cell types^86,87^. Recent studies have demonstrated that excessive zinc disrupts the homeostasis of ER and mitochondrial function, resulting in impaired cell function and viability. For example, zinc decreases intracellular ATP levels and hampers the viability of MIN6 insulinoma cells^23^. Moreover, cumulative evidence illuminates that excessive zinc induces ER stress and gives rise to neuronal dysfunction and death^88–90^. Recently, it was reported that elevated zinc in mitochondria leads to mitochondrial fission and β cell death under PA treatment^91^. Furthermore, zinc accumulation by mitochondrial zinc exporters impairs mitochondrial structure and function, negatively affecting animal development and lifespan in *Caenorhabditis elegans*^92^. Moreover, previous studies demonstrated that co-secreted zinc not only plays an inhibitory role in insulin secretion^35–38^, but also acts as a paracrine death effector for pancreatic β cells^58,59^. Consistent with a previous study, we found that ZnT8 LOF dramatically decreases granular zinc, mitigates ER stress, alleviates mitochondrial depolarization and decreases cell death within SC-β cells under metabolic stress. However, how the decrease in granular zinc induced by ZnT8 LOF alleviates ER and mitochondrial dysfunction remains to be further investigated.

Human *SLC30A8* variants, such as haploinsufficient ZnT8 p. Arg138X, and the missense polymorphism of ZnT8 p. Trp325Arg, are strongly associated with the risk of diabetes in humans. The differential molecular mechanisms underpinning the seemingly irreconcilable phenotypes in diabetes pathogenesis have yet to be disentangled. The WT *SLC30A8* p. R325 variant^93,94^ has long been considered to possess zinc transportation activity inferior to that of the p. W325 variant (in which arginine is replaced by tryptophan at position 325 of ZnT8)^12,95^ and is associated with a higher risk of diabetes than the p. W325 variant^1,22^. Nevertheless, this conclusion was based on ectopic overexpression of the human p. R325 and p. W325 variants by plasmid transfection or adenovirus infection in either a mouse or rat tumor cell line^12,95^. Recently, an elaborate biochemical study with purified ZnT8 variant proteins showed that the common high-risk p. R325 variant is hyperactive and exhibits accelerated zinc transport kinetics compared with the p. W325 variant associated with a lower risk^96^, which contradicts previous conclusions from rodent tumor cell lines, but is consistent with human genetic results that the rare loss of function ZnT8 mutations decreases the risk of diabetes. However, more evidence is still needed to make a solid conclusion on the comparison of zinc transport between the p. R325 and p. W325 variants in human models. Thus, a more rigorous analysis of gene-edited SC-β cells with p. R325 and p. W325 warrants further investigation. Nevertheless, ZnT8 stands as a promising target for SC-β cell therapy.

## Methods

### Ethics statement

All experiments performed in this study were compiled with ethical regulations and approved by the Biological Research Ethics Committee of Tongji University. The details are described in the respective sections below.

### Maintenance of hESCs

hESCs were cultured on irradiated mouse embryonic fibroblasts (iMEFs), which were seeded on the dishes coated with 1:6 diluted Matrigel (Corning, #354277) with hESC medium. This medium contains DMEM/F12 (Gibco, #11330-082), 20% Knockout Serum Replacement (KOSR) (Gibco, #10828028), 1 mM non-essential amino acids (Gibco, #11140-050), 1% L-glutamine (Gibco, #25-005-CI), 0.1 mM β-mercaptoethanol (Gibco, #21985023), and 10 ng ml^−1^ bFGF (R&D System, #233-FB/CF). Cells were maintained at 37 °C with 5% CO_2_ and passaged every 3–4 days by using TrypLE (Gibco, #12604013) for dissociation.

### Generation of mutant cell lines

The HUES8 hESCs were provided by Dr. Qiurong Ding. MEL1* INS*^*GFP/W*^ hESCs^27^ were provided by Dr. E. Stanley and Dr. A. Elefanty. The information for gene-edited cell lines, sgRNA sequences, primers for qPCR, and antibodies used in this study are listed in Supplementary Tables 1–4.

*Generation and identification of targeted MEL1 NKX6.1*^*mCherry/mCherry*^*-*INS^GFP^^*/W*^ *hESCs*. The *NKX6.1*^*mCherry/mCherry-*^*-*INS-GFP^*/W*^ MEL1 knock-in cell line was generated by inserting sequences encoding T2A and mCherry into the two alleles of the *NKX6.1* locus of INS^*GFP/W*^ MEL1 hESCs using CRISPR/Cas9. The Px330 vector^97^ expressing Cas9 and sgRNA was kindly provided by Jingsong Li. A mCherry selection cassette was inserted into the vector for single-cell sorting. The donor vector was generated by inserting homologous left arm-T2A-mCherry-homologous right arm into pMD18-T vector (TaKaRa, #6011). *INS*^*GFP/W*^ MEL1 hESCs were transfected with the Px330 vector and donor plasmid using Fugene HD (Promega, #E2311) within 24 h after passage. Transfected single cells were sorted by mCherry after transfection for 24 h and seeded on iMEF-coated dishes. Single-cell-derived clones were picked for genotyping 7–9 days later.

*Generation and identification of SLC30A8*^*−/−*^ *and INS*^*−/−*^ *hESC lines*. The sgRNA targeting exon 4 of the human *SLC30A8* gene was designed (www.genome-engineering.org/crispr) and inserted into the vector carrying a Cas9 gene and a puromycin resistance cassette. The construct (1 μg) was electroporated into 0.4 million hESCs and seeded on 10 cm plates with hESC medium. Puromycin was used to select the cells that successfully transfected the plasmid. 72 h later, puromycin was removed and the medium was changed every day until the colonies were visible. A portion of each clone was lysed and analyzed by Sanger sequencing and the remaining portion was cryopreserved. For *INS*^*−/−*^ hES cell line, the sgRNA targeting exon1 of human *INS* gene was designed using the CRISPR on-line design tool (www.genome-engineering.org/crispr) and inserted into the vector. The methods of transfection in the MEL1 *NKX6.1*^*mCherry/mCherry*^*-*INS^GFP^^*/W*^ hESCs as described above.

### In vitro differentiation of SC-β cells derived from hESCs

hESCs were differentiated into SC-β cells using a protocol adapted from a previous study^9,98^.In brief, hESCs were cultured on the Matrigel-coated (1:3 diluted) plate to start differentiation until 90% confluency. The differentiation process included planar culture through stage 1 to stage 4 to generate NKX6.1^+^ progenitors at high efficiency and 3D culture from stage 5 to stage 7 to generate mature SC-β-cells (See details in Supplementary Table 5).

### Quantitative real-time PCR

Total RNA was isolated using the TianGen TRNzol Universal kit (TianGen, #DP424) following the manufacturer’s instructions. First strand cDNA was generated using the TianGen Quantscript RT Kit (TianGen, #KR106) and the products were used as qRT-PCR templates in the SYBR Green-based qPCR. Triplicate reactions (technical replicates) were carried out for each biological replicate. GAPDH was used as a housekeeping control to normalize targeted gene expression.

### Immunofluorescence staining

#### For adherent cells

Cells were washed once with precooled PBS and fixed with 4% paraformaldehyde for 15 min at room temperature (RT). After being washed with PBS, the cells were permeabilized with PBST (PBS with 0.1% TritonX-100) for 15 min and then blocked with PBST containing 5% donkey serum (blocking solution) for 2 h. The samples were incubated with the primary antibody diluted with blocking solution overnight at 4 °C. After being washed with PBS three times, the samples were incubated with Alexa Fluro secondary antibodies for 1 h at RT and cell nuclei were stained with DAPI dihydrochloride. Images were taken using Leica SP8 and ZEISS LSM880 confocal microscopes.

#### For frozen tissue sections

The fixed SC-β cell clusters or tissues (dehydrated) were embedded in optimum cutting temperature (OCT) and stored at −80 °C for analysis using cryostat sectioning. The sections were sliced to 8 µm on the slice with a cryostat microtome and incubated with a blocking solution for 2 h at RT. The slices were stained with the antibodies as described above.

### Flow cytometry

For intracellular staining, the SC-β cell clusters were dissociated into single cells, fixed with 1.6% PFA at 37 °C for 20 min and permeabilized with 1:10 diluted saponin buffer (Biolegend, #421002) for 5 min. After washed with PBS, the cells were incubated with primary antibodies and fluorescence-conjugated secondary antibodies for 30 min at RT. The cells were then washed and resuspended in FACS buffer (PBS containing 0.5% BSA) for flow cytometry analysis by BD FACSVerse and Moflo Astrios 4 lasers. Data were analyzed by using FlowJo v10.

### Zinquin staining

SC-β cell clusters or adherent cells were washed once with FACS buffer and subjected to the Zinquin dye (20 nM, Sigma-Aldrich, #Z2251) for 20 min at 37 °C and 5% CO_2_. Cells stained with Zinquin were observed immediately by using a ZEISS microscope with emission at 482–488 nm and excitation at 361–367 nm.

### Insulin secretion assays

#### Static insulin secretion assay

Before stimulation, SC-β cell clusters needed to be starved in Krebs-Ringer buffer^9^ supplemented with 2 mM glucose for 2 h in a 37 °C, 5% CO_2_ incubator. For glucose stimulation, the SC-β cells were stimulated alternately by Krebs-Ringer buffer with low (2 mM) or high (20 mM) glucose. Supernatants were collected after 30 min of each stimulation and the pellets were lysed overnight in acidified alcohol (75% alcohol, 1.5% HCl) at −20 °C for insulin content measurement. The same procedures were carried out for treatments with 30 mM KCl, 200 μM tolbutamide, or 10 nM exendin-4. Insulin (secreted or content) was measured by a human insulin ELISA kit (ALPCO, #80-INSHU-E01.1).

#### Dynamic perifusion assay

SC-β cell clusters were handpicked, washed with Krebs-Ringer buffer twice, and suspended in 2 mM glucose Krebs-Ringer buffer. Then clusters were loaded onto each chamber of an automated Biorep Perifusion System between two layers of Bio-Gel P-4 polyacrylamide beads. Under temperature-controlled conditions, clusters were perfused at a flow rate of 100 μl min^−1^ and samples were collected with 1 min collection points. Prior to sample collection, clusters were equilibrated under basal (2 mM glucose) conditions for 45 min. Then, solutions were switched as follows: 2 mM glucose for 10 min, 20 mM glucose challenge for 30 min, 10 nM Extendin-4 with 20 mM glucose for 10 min, 2 mM glucose for 10 min, and 30 mM KCl for 10 min. For the zinc inhibition assay, 100 μM ZnSO_4_ was added during the high glucose challenge for 30 min. For normalization, DNA content was determined by KAPA extraction and a Quant-iT PicoGreen dsDNA Kit (Invitrogen, #P7589).

#### Proinsulin to C-peptide conversion analysis

The SC-β cell clusters were lysed overnight in acidified alcohol at −20 °C after 2 h starvation in 2 mM glucose Krebs-Ringer buffer. The supernatants were used to measure proinsulin and C-peptide levels using a human proinsulin ELISA kit (Mercodia, #1118-1-10) and a human ultrasensitive C-peptide ELISA kit (Mercodia, #0111-1-10).

### Electron microscopy

SC-β cell clusters were spun down to remove excess medium and washed with PB (PBS without sodium chloride) two times, followed by the addition of ice-cold fixative (2.5% glutaraldehyde) overnight. The fixed clusters were further processed using an electron microscopy sample generation protocol by the Electron Microscopy Facility at the School of Life Sciences and Technology, Tongji University. Pictures of insulin granules were taken by a JEM-1230 transmission electron microscope.

### Single-cell RNA sequencing

#### Alignment

A 16-bp cell barcode and 9-bp unique molecular identifier (UMI) were contained in read 2 of our paired-end sequencing data, while all genomic information was contained in read 1. We used the Drop-seq pipeline to perform the alignment (version 1.13)^99^. Briefly, we added the barcode and UMI information into the tag and trimmed adapter, 5’ primer and 3’ polyA from the reads. Then the trimmed reads were aligned to the human genome with transcriptome annotation (GRCh38, ensemble 93) using STAR (version 2.6.0a)^100^. After that, the tags were merged with the aligned SAM file. The barcode substitution and indel errors were repaired. Finally, a digital gene expression matrix was obtained from barcode-UMI-gene triplets. The remaining cell barcodes were consistent with those we used as input. Scater was used to filter out the low-quality cells^101^. Cells with library sizes greater than 2000 UMI counts, with a number of detected genes greater than 1000 and a percentage of mitochondrial genes <25% were used for subsequent analysis. In total, 1543 cells passed the criteria, while 89 cells failed.

#### Cell clustering

Seurat (version 2.3.4) was used to identify cell clusters^102^. First, the UMI count matrix was used as input, and genes expressed in at least 2 cells were kept, leaving 25426 out of 31097 genes for downstream analysis. UMI counts were normalized and scaled by the default parameters. Genes with an average expression between 0.0125 and 5 and dispersion greater than 0.25 were considered variable genes. Then, variable genes were used as input to perform principal component analysis (PCA). The Jackstraw procedure was used with 1000 replicates to identify significant PCs with a strong enrichment of differences to separate the cells. PCs 1–12 were selected to run t-SNE and perform the clustering through the FindClusters function. Pseudotime analysis was performed only on S6WT, S6KO, S7WT, and S7KO cells, using Monocle (version 2.0) with default parameters^103,104^.

#### Differential expression analysis, GO enrichment and GSEA

To identify DEGs between two clusters, we used the Seurat function FindMarkers with the following parameters: logfc. threshold = 0.25, test.use = “wilcox”, min.pct = 0.1. The cell clusters used for the comparisons were those four clusters corresponding to four given cell types. Genes with adjusted *p value* no more than 0.05 were selected. Volcano plots and Venn diagrams were plotted using R. Heatmap and violin plots were plotted using Seurat. GO enrichment was performed using ClusterProfiler (version 3.10.0) with the following parameters (ont = “BP”, pAdjustMethod = “BH”, pvalueCutoff = 0.01, qvalueCutoff = 0.05). GSEA was performed using ClusterProfiler (version 3.10.0) with default parameters, except minGSSize changed to 100^105^.

### Annexin V/PI apoptosis analysis

SC-β cells were incubated with PA (Sigma-Aldrich, #P0500), SA (Sigma-Aldrich, #S4751) and LA (Sigma-Aldrich, #l5900) or solvent control (8% BSA in 0.1 M NaOH for PA and SA; ddH2O for LA) in albumin-free medium for 12–24 h^40–42,106^ and dissociated into single cells by 0.25% trypsin. Cell death was measured by using an Annexin V/PI apoptosis detection kit (YEASEN, 40304ES60) and assessed by flow cytometry.

### Western blot

Total proteins were obtained by treating the SC-β cells in RIPA lysis buffer. To obtain equal amounts of protein, the bicinchoninic acid (BCA) protein quantification kit (YEASEN, #20201ES76) was used to measure the protein concentrations. The proteins were separated on 10% SDS/PAGE gels and transferred to nitrocellulose membranes (PALL, #66485). The blots were visualized by enhanced chemiluminescence (ECL) and exposed to an Amersham Imager 600. The uncropped and unprocessed scans of the blots were in the Source Data file.

### Cytosolic calcium measurement

The HUES8 SC-β cell clusters were dissociated into single cells by 0.25% trypsin and seeded onto 3.5 cm Matrigel-coated glass-bottom petri dishes. 24 h later, the SC-β cells were stained with Fluo-4 AM (Invitrogen, #F14201, 2 μM) at 37 °C for 30 min. Consecutive images of the treated cells were taken every 2 s by confocal microscopy. The fluorescence intensity of Fluo-4 was measured by circling the entire single cell with Image J. The trace was plotted according to relative fluorescence intensity (F_1_/F_0_; F_1_, the fluorescence intensity of each image; F0, the fluorescence intensity of the first image).

### Mitochondrial membrane potential measurement

The dissociated HUES8 SC-β cells were seeded onto 3.5 cm Matrigel-coated glass-bottom petri dishes for 24 h and loaded with JC-10 (Abcam, #ab112134, 10 mg ml^−1^) for 1 h. During the period of JC-10 staining, several dishes of HUES8 SC-β cells were treated with FFAs in parallel for 0 min or 30 min or 45 min, or 60 min, respectively. A confocal microscope was used to take images with the excitation = 540 nm, emission = 590 nm for aggregated imaging, and excitation = 490 nm, emission = 525 nm for monomeric imaging. The mitochondrial membrane potential was measured by the ratio of green monomeric to red aggregated fluorescence.

### Mouse studies

Male SCID-Beige mice were obtained from Beijing Vital River Laboratory Animal Technology Company and maintained in the animal facility of Tongji University, Shanghai, China. All experiments were performed in accordance with the University of Health Guide for the Care and Use of Laboratory Animals and approved by the Biological Research Ethics Committee of Tongji University. The mice aged 6–8 weeks were fed with the chow for immunodeficient mice (XieTong ShengWu, #1010019) and housed in a constant temperature room (22 ± 1 °C) with a 12-h light/dark cycle and 40–60% humidity.

#### SC-β cells transplantation in STZ-induced diabetic mice

Mice with diabetes were generated by a single injection of streptozotocin (STZ, Sigma-Aldrich, #S0130) (170 mg kg^−1^) and then transplanted with the same amount of WT or *SLC30A8*^*−/−*^ SC-β cells (3 million per animal) under the left kidney capsule. The nontransplantation diabetic mice were used as controls. Body weight and randomly fed blood glucose were measured with a handheld glucometer following transplantation. Glucose-stimulated human insulin secretion was measured by collecting mouse serum from the eye socket after 16 h of fasting and at 25 min after glucose intraperitoneal injection (3 g kg^−1^, 30% solution). For the glucose tolerance assay, the mice were fasted for 16 h and injected with glucose (3 g kg^−1^, 30% solution). At the time points of 0, 15 min, 30 min, 60 min and 120 min, the blood glucose of mice was monitored by using a tail bleed.

#### SC-β cells transplantation in non-STZ-induced healthy mice

Same amount of WT or *SLC30A8*^*−/−*^ SC-β cells (three million cells per animal) were transplanted into healthy SCID-Beige mice under the kidney capsule. Glucose-stimulated human insulin secretion was performed as described above. The proinsulin and C-peptide levels were also measured within the mice serum, which was collected after 16 h of fasting.

### Statistical analysis

Data were derived from at least three independent biological replicates. Mean ± s.e.m. was used to present quantification data. *p* values were calculated by two-tailed unpaired Student’s *t* test if not otherwise specifically indicated. For multiple comparisons, *p* values were calculated by one-way analysis of variance (ANOVA) or two-way repeated-measures ANOVA. *p < 0.05, **p < 0.01, ***p < 0.001, ****p < 0.0001. ns, not significant.

### Reporting summary

Further information on research design is available in the Nature Research Reporting Summary linked to this article.

## Supplementary information

Supplementary Information

Reporting Summary

## Data Availability

The single-cell RNA-seq data of this study have been deposited in the Gene Expression Omnibus (GEO) under accession code GSE135076. The data supporting the findings of this study are available from the corresponding authors on request. Source data are provided with this paper.

## Source data

Source Data
