# Supplementary material for: ZnT8 loss-of-function accelerates functional maturation of hESC-derived β cells and resists metabolic stress in diabetes
Source: Nat Commun. 2022 Jul 16;13:4142. doi: 10.1038/s41467-022-31829-9 (PMC9288460; doi:10.1038/s41467-022-31829-9)
Supplement: Supplementary file 1 — Supplementary Information [file 41467_2022_31829_MOESM1_ESM.pdf]

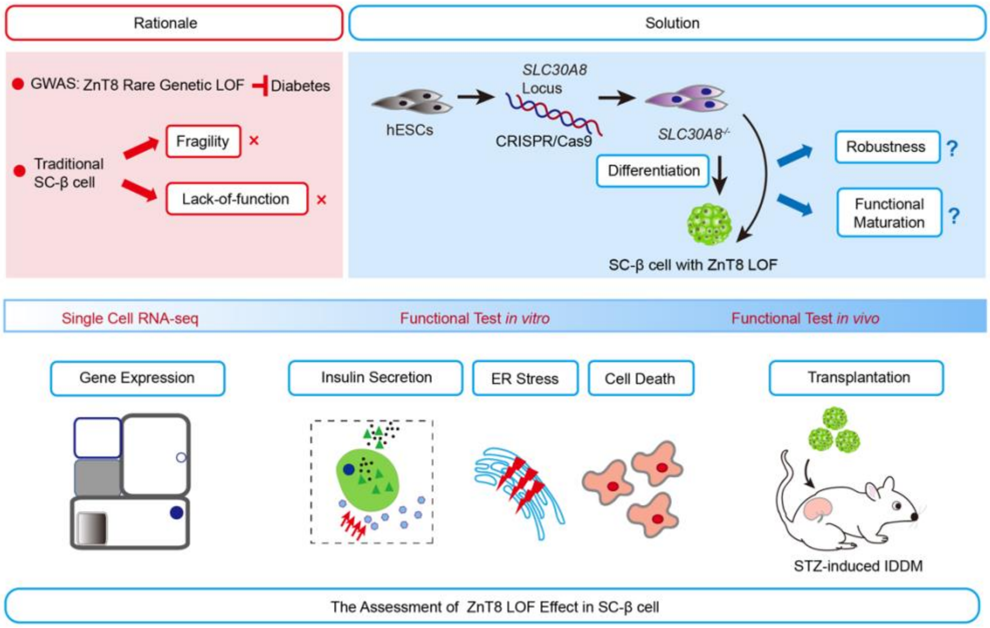

Supplementary Fig. 1, A flowchart describing the study design. A study design of the rationale, the solution, and the assessment of SC-β cells with ZnT8 LOF including gene expression, *in vitro* function tests, and *in vivo* function tests. GWAS, genome-wide association study; LOF, loss-of-function; IDDM, insulin-dependent diabetes mellitus; SC-β cell, human embryonic stem cell (hESC) derived β cells; ER, endoplasmic reticulum.

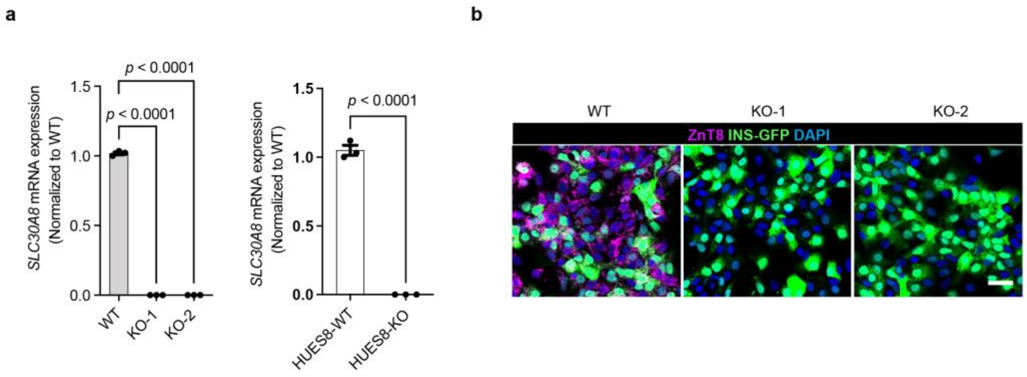

Supplementary Fig. 2, Validation of *SLC30A8* knockout. a, qRT-PCR analysis of *SLC30A8* expression in the cell lines used in this study (WT, KO-1, KO-2, HUES8-WT and HUES8-KO) (One-way ANOVA with Dunnett's test for MEL1, unpaired two-tailed *t*-tests for HUES8, $n = 3$). **b**, Representative immunofluorescent staining of ZnT8 in WT, KO-1 and KO-2 adherent SC-β cells. Scale bar, 25 μm. $n = 3$ independent experiments. Data in this figure are presented as mean ± s.e.m. Individual data points are shown for all bar graphs. Source data are provided as a Source Data file.

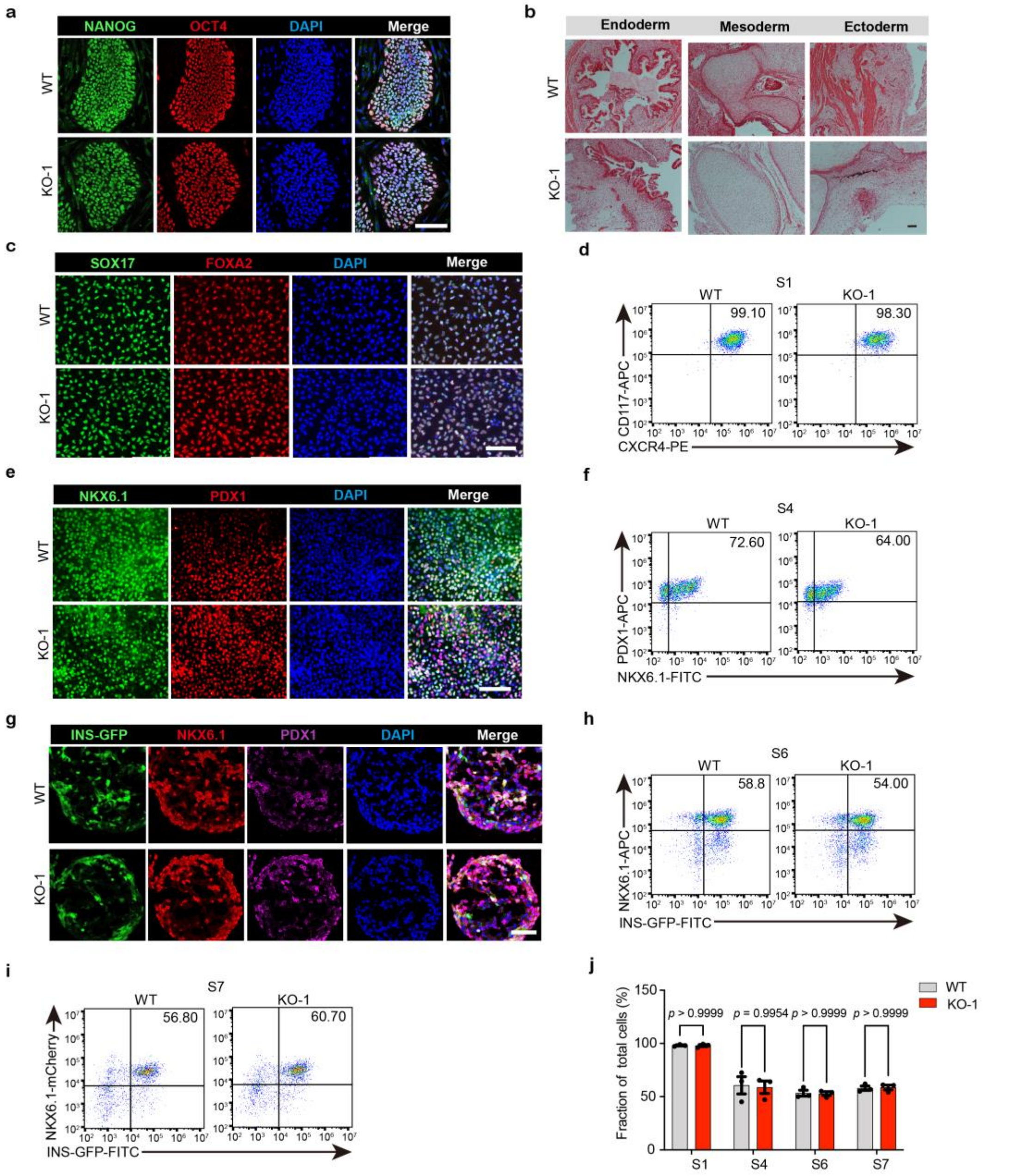

Supplementary Fig. 3, Differentiation efficiency is not affected by ZnT8 LOF in the MEL1 hESCs. a, Representative immunostaining of NANOG and OCT4 in WT and KO-1 hESCs. Scale bar, 100 μm. **b**, Hematoxylin and eosin staining showing teratoma formation after WT and KO-1 hESCs were transplanted subcutaneously into SCID-Beige mice for 6 weeks. The staining showed typical teratomas containing ectoderm, mesoderm and endoderm derived tissues. Scale bar, 100 μm. **c**, **e**, **g**, Representative immunostaining of SOX17 and FOXA2 in WT and KO-1 S1 cells (**c**); NKX6.1 and PDX1 in S4 cells (**e**); and INS-GFP, NKX6.1 and PDX1 in S6 cells (**g**). Scale bars, 100 μm. **d**, **f**, **h**, **i**, Representative FACS plots showing the percentage of CD117^{+}/CXCR4^{+} in S1 cells (**d**), PDX1^{+}/NKX6.1^{+} in S4 cells (**f**), NKX6.1^{+}/INS-GFP^{+} in S6 cells (**h**) and NKX6.1-mCherry^{+}/INS-GFP^{+} in S7 cells (**i**). **j**, FACS quantifications of the key markers of S1, S4, S6 and S7 in WT and KO-1 cells (two-way ANOVA with

Sidak's test for multiple comparisons, $n = 3$). $n = 3$ independent experiments in the **a, b, c, e, g.** Data in this figure are presented as mean ± s.e.m. Individual data points are shown for all bar graphs. Source data are provided as a Source Data file.

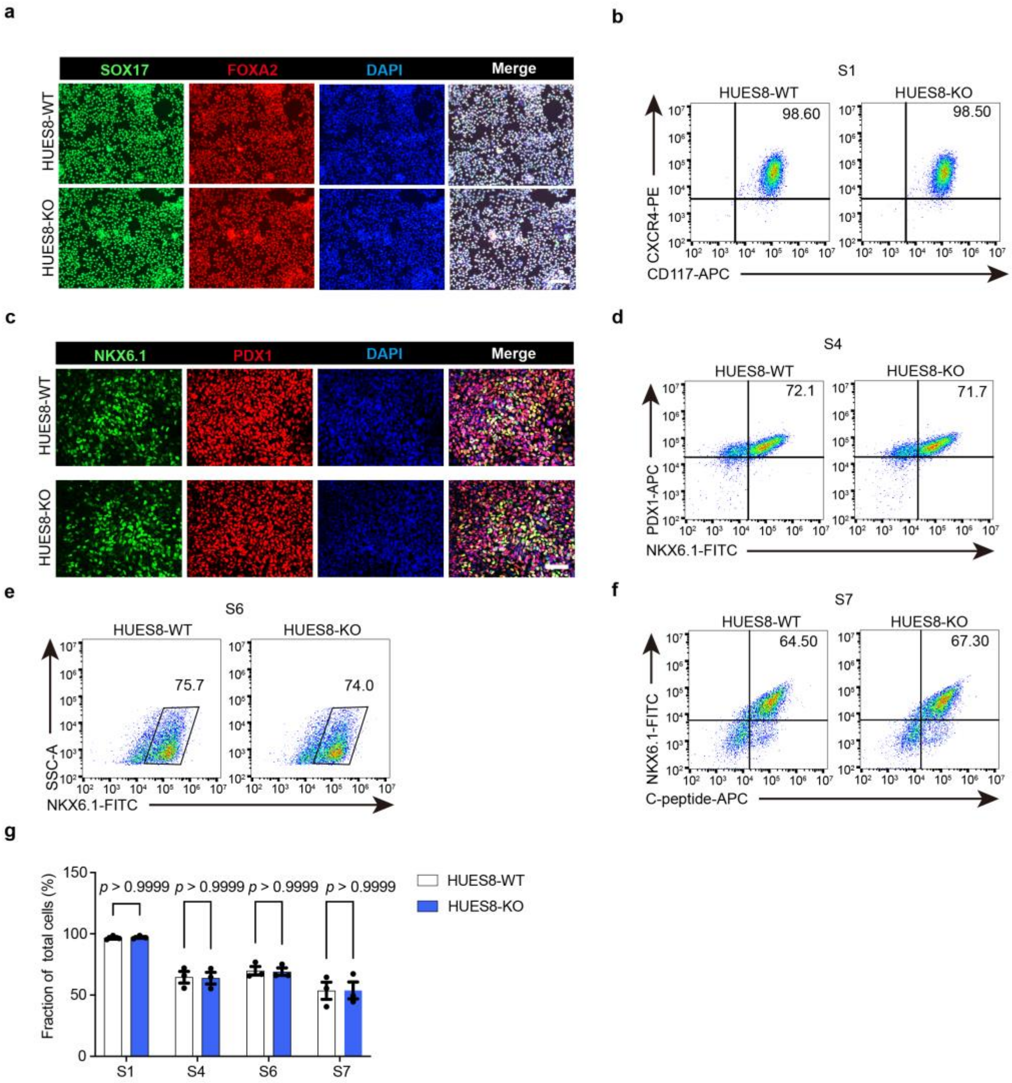

Supplementary Fig. 4, Differentiation efficiency is not affected by ZnT8 LOF in the HUES8 hESCs. a, Representative immunostaining of SOX17 and FOXA2 in WT and KO-1 S1 cells. Scale bar, 100 µm. **b**, Representative FACS plots showing the percentage of $CXCR4^+/CD117^+$ in S1 cells. **c**, Representative immunostaining of NKX6.1 and PDX1 in WT and KO-1 S4 cells. Scale bar, 50 µm. **d**, Representative FACS plots showing the percentage of $PDX1^+/NKX6.1^+$ in S4 cells. **e**, Representative FACS plots showing the percentage of $NKX6.1^+$ in S6 cells. **f**, Representative FACS plots showing the percentage of $NKX6.1^+$/C-peptide$^+$ in S7 cells. **g**, FACS quantifications of S1, S4, S6 and S7 in HUES8-WT and HUES8-KO cells (two-way ANOVA with Sidak's test for multiple comparisons, $n = 3$). $n = 3$ independent experiments in the **a and c.** Data are presented as mean ± s.e.m. Individual data points are shown for all bar graphs. Source data are provided as a Source Data file.

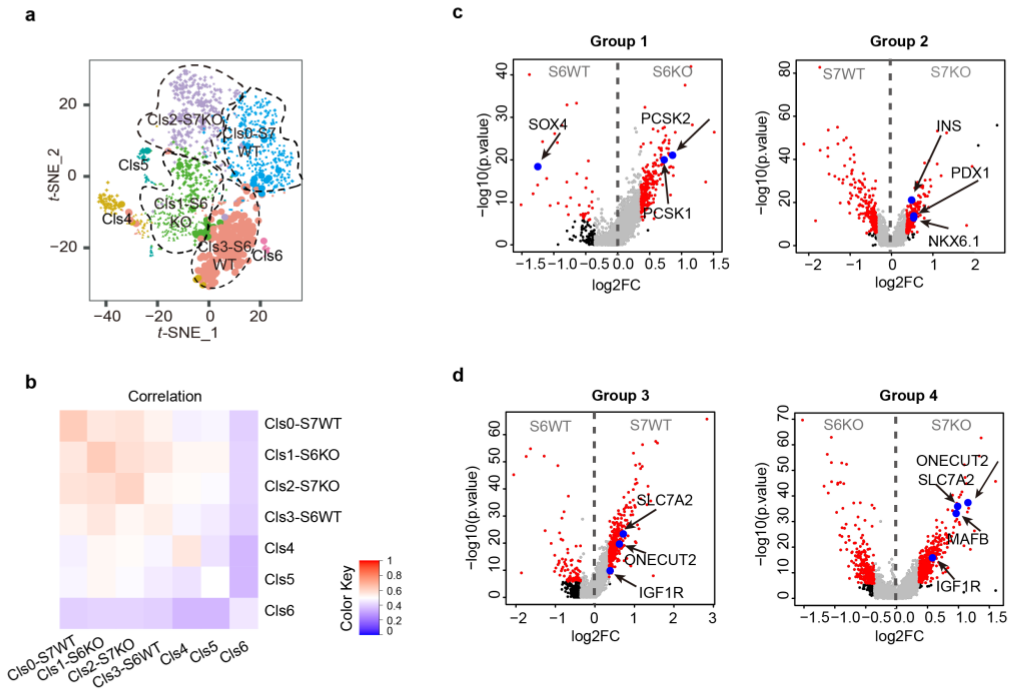

Supplementary Fig. 5, *t*-SNE projection of the all clusters analyzed by single-cell RNA-seq. **a**, *t*-SNE projection of Cls0-S7 WT, Cls1-S6KO, Cls2-S7KO, Cls3-S6WT, and Cls4-6 colored by Seurat identified clusters (cls, cluster). **b**, The heatmap of mean Spearman correlation coefficient between clusters. **c**, **d**, Volcano plots of the up-regulated and down-regulated genes (DEGs) according to the comparison of each group. Genes with absolute fold change not more than 1.28 are in grey, genes with absolute fold change greater than 1.28 and adjusted p value not more than 0.05 are in red, and others are in black.

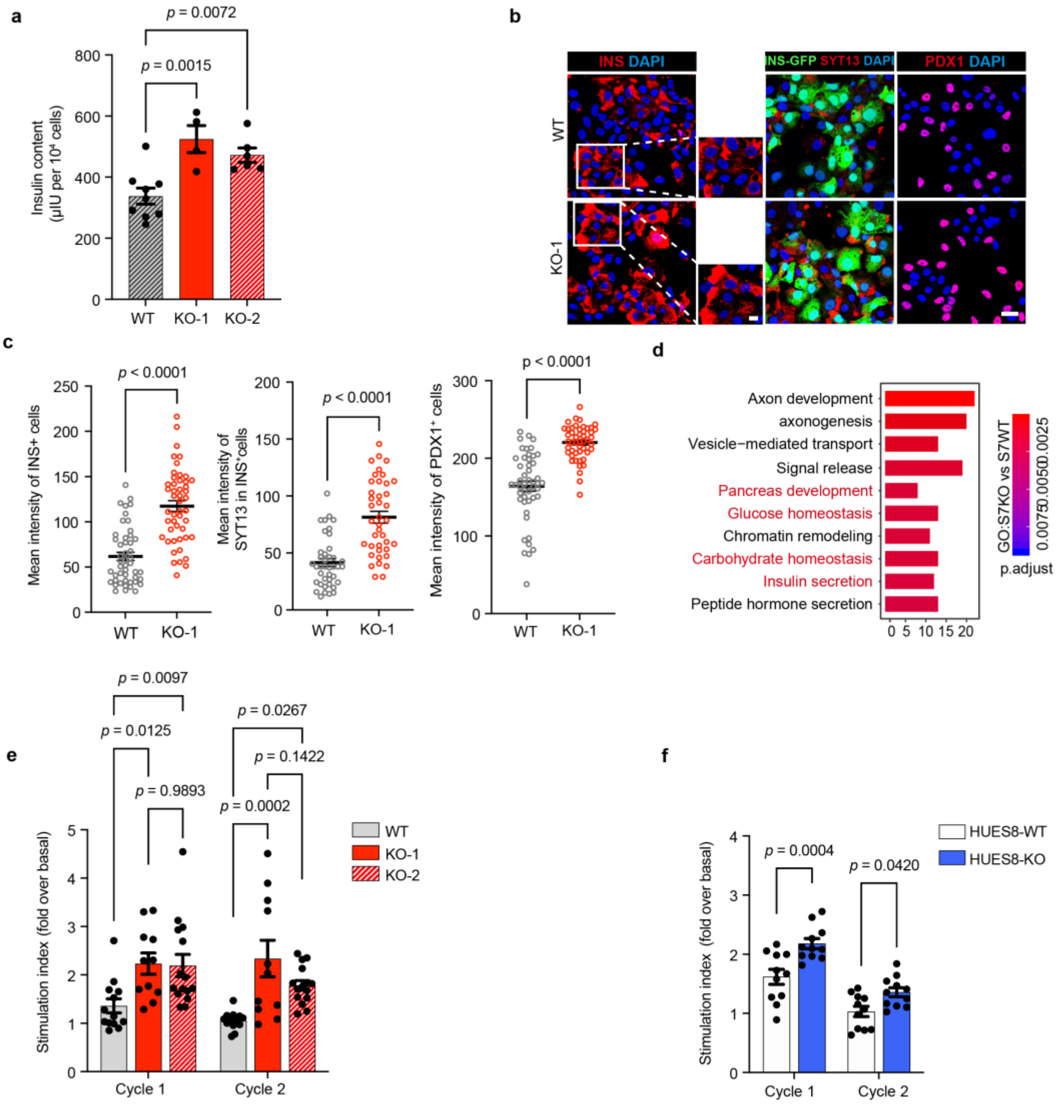

Supplementary Fig. 6, Additional analysis of ZnT8 LOF promoting SC-β cell maturation. a, Insulin content of S7 pure SC-β cells (WT, n = 9; KO-1, n = 4; KO-2; n = 6; one-way ANOVA with Dunnett's test for multiple comparisons). **b**, **c**, Representative immunofluorescent images (**b**) and mean intensity measurements (**c**) of INS, SYT13 and PDX1 in WT and KO-1 adherent SC-β cells (unpaired two-tailed *t*-tests; INS, n = 48; SYT13, n = 40; PDX1, n = 48; data points are from 5 independent experiments for each condition). Scale bar in low magnification, 25 µm; Scale bars in high magnification, 5 µm. **d**, Ten enriched GO terms in KO cells relative to WT SC-β cells. The red-colored terms represent β cell function-related processes. **e**, **f**, Data is expressed as the fold change relative to basal insulin levels of Fig. 2b (**e**; WT, n = 13; KO-1, n = 11; KO-2, n = 15; two-way ANOVA with Tukey's test for multiple comparisons) and Fig. 2c (**f**; n = 11; two-way ANOVA with Sidak's test for multiple comparisons). Data are presented as mean ± s.e.m. Individual data points are shown for all bar graphs. Source data are provided as a Source Data file.

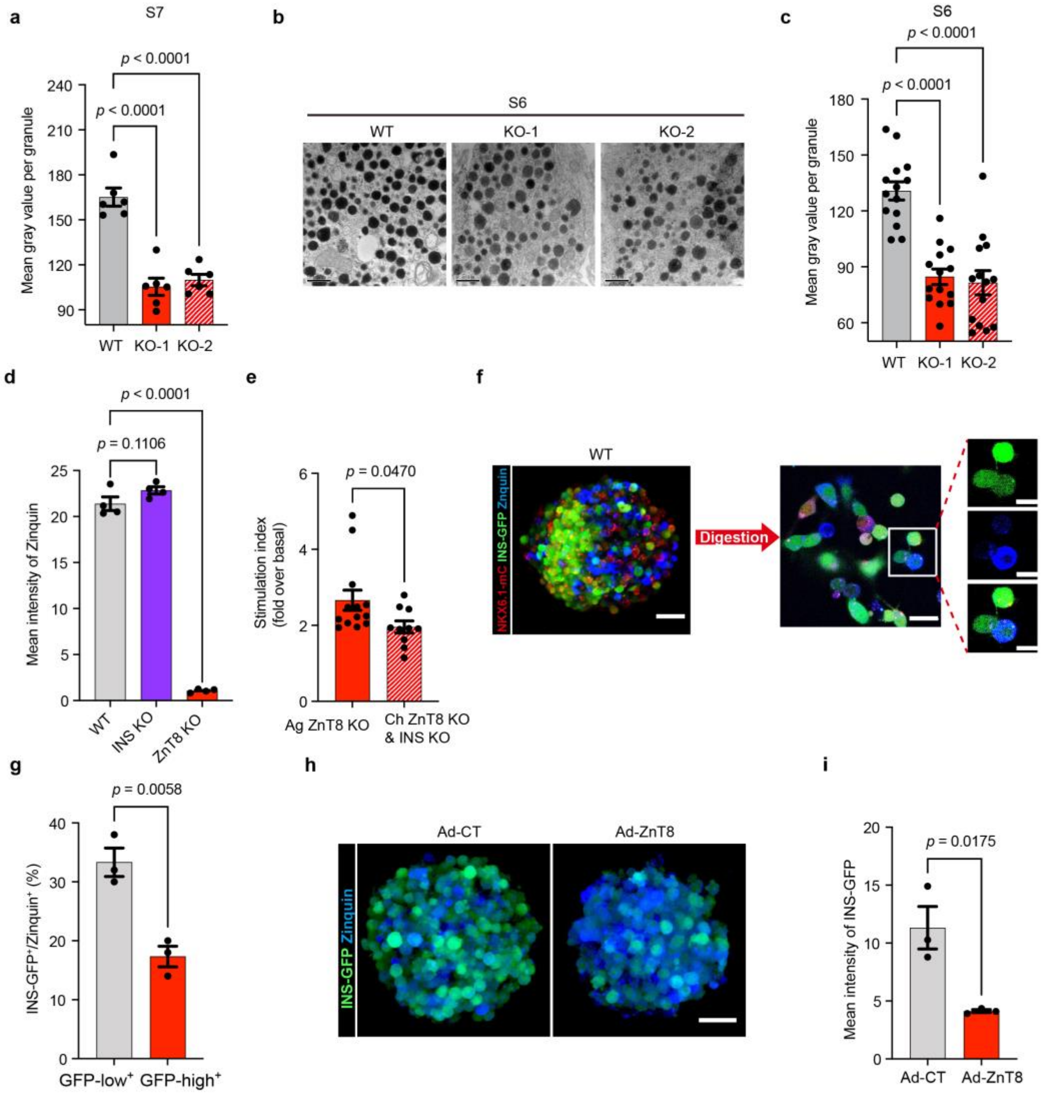

Supplementary Fig. 7, Additional analysis of zinc inhibition on insulin secretion. **a**, Mean gray value measurements of insulin granules in WT, KO-1 and KO-2 S7 SC-β cells (n = 6). The images are shown in Fig. 3b. **b**, **c**, Transmission electron micrographs (**b**) and quantifications (**c**) of immature insulin granules in WT and KO-1, KO-2 SC- β cells of S6 (n = 14). **d**, Mean intensity of Zinquin in WT, INS KO and ZnT8 KO SC-β cells (n = 4). The images are shown in Fig. 3g. **e**, Data is expressed as the fold change relative to basal insulin levels shown in Fig. 3j. (Aggregated ZnT8 KO, n = 13; Chimera ZnT8 KO & INS KO, n = 10) **f**, Fluorescence images of NKX6.1-mCherry, INS-GFP and Zinquin (Zn^{2+}) in WT SC-β cells. Left panel: 3D reconstruction confocal image; scale bar, 50 μm. Right panel: images of adherent single cells dissociated from SC-β cells clusters; scale bar in low magnification images, 20 μm; scale bar in high magnification images, 10 μm. **g**, Percentage of INS-GFP$^+$ cells in Zinquin$^+$ cells. GFP-high$^+$ cells represent SC-β cell with strong GFP intensity (n = 3); GFP-low$^+$ cells represent SC-β cells with weak GFP intensity (n = 3). GFP signal intensity was measured by ImageJ. **h**, **i**, Zinquin staining (**h**) and mean intensity measurements of INS-GFP (**i**) in WT SC-β cells infected with Ad-CT (Adenovirus-

Control) and Ad-ZnT8 (Adenovirus-ZnT8). Images shown are 3D confocal re-construction with maximum intensity projections (n = 3). Scale bar, 50 μm. One-way ANOVA with Dunnett's test for **a**, **c** and **d**, unpaired two-tailed *t*-tests for **e**, **g** and **i**. Data are presented as mean ± s.e.m. Individual data points are shown for all bar graphs. Source data are provided as a Source Data file.

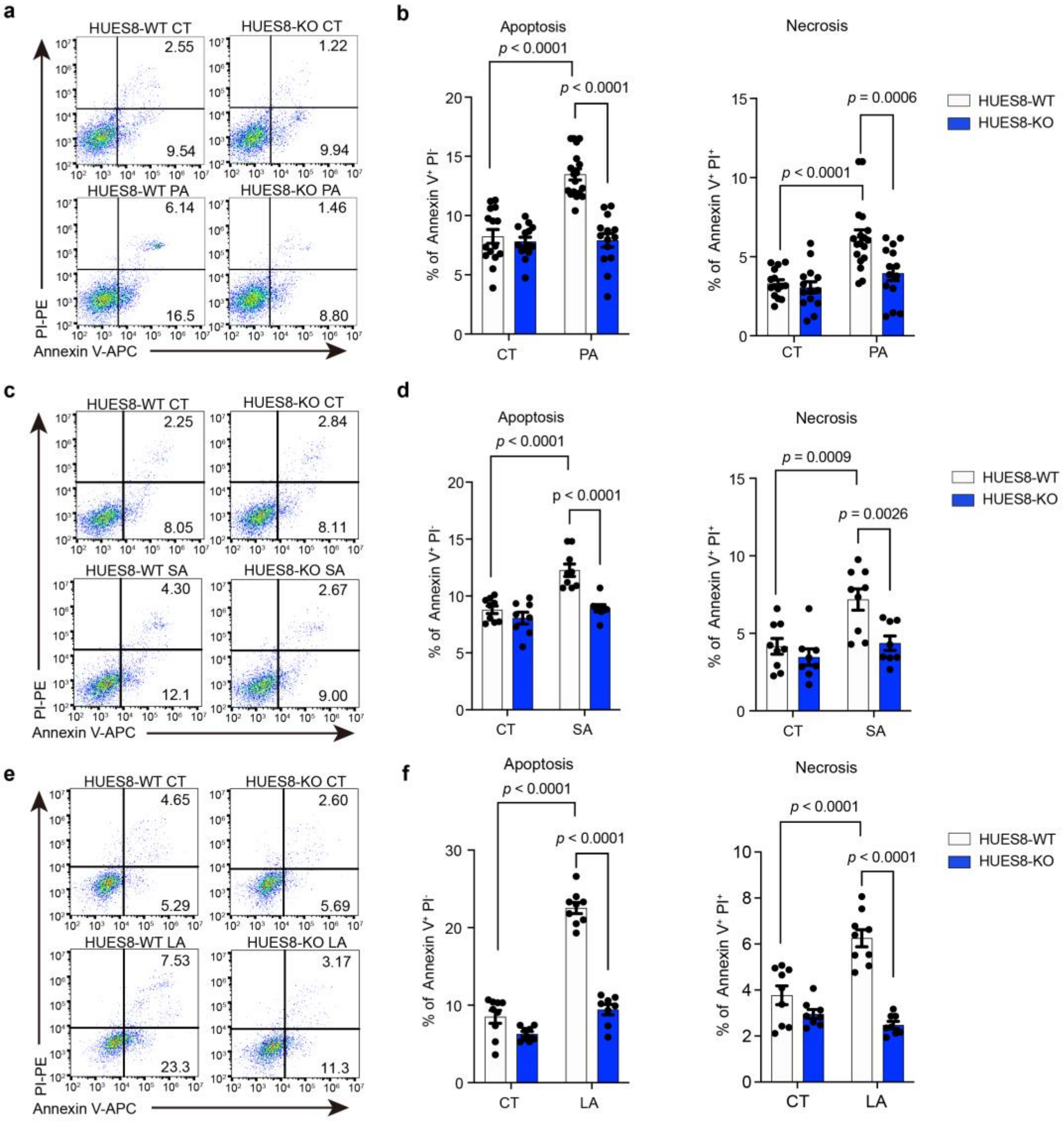

Supplementary Fig. 8, Additional analysis of SC-β cell with ZnT8 LOF resistance to lipotoxicity. a, **b**, Representative FACS plots **(a)** and quantifications (**b**) of apoptosis (Annexin V$^+$/PI$^-$) and necrosis (Annexin V$^+$/PI$^+$) populations in HUES8-WT and HUES8-KO SC-β cells treated with PA or solvent control for 24 h (HUES8-WT CT, *n* = 15; HUES8-WT PA, *n* = 18; HUES8-KO, *n* = 14). **c**, **d**, Representative FACS plots (**c**) and quantification (**d**) of apoptosis and necrosis in HUES8-WT and HUES8-KO SC-β cells treated with SA or solvent control for 24 h (HUES8-WT, *n* = 9; HUES8-KO, *n* = 8). **e**, **f**, Representative FACS plots (**e**) and quantification (**f**) of apoptosis and necrosis in HUES8-WT and HUES8-KO SC-β cells treated with LA or solvent control for 24 h (HUES8-WT, *n* = 9; HUES8-KO, *n* = 8). Two-way ANOVA with Sidak's multiple-comparisons test was used for analysis for **b**, **d** and **f**. Data in this figure are presented as mean ± s.e.m. Individual data points are shown for all bar graphs. Source data are provided as a Source Data file.

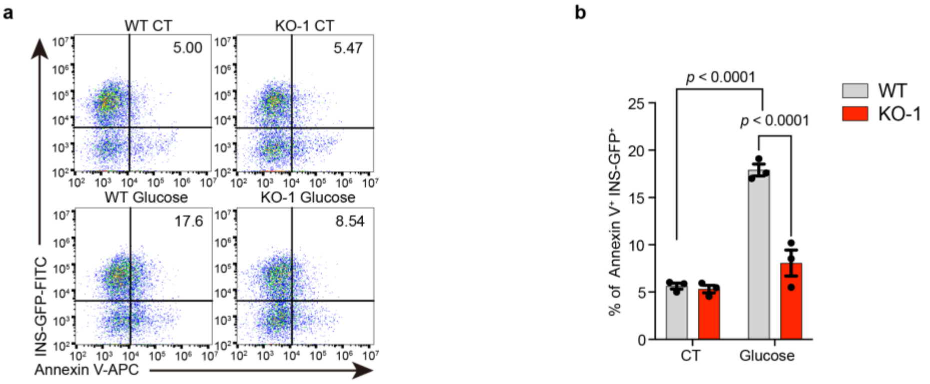

Supplementary Fig. 9, ZnT8 LOF SC-β cells are resistant to glucotoxicity-triggered cell death. **a**, **b**, Representative FACS plots (**a**) and quantification (**b**) of Annexin V^+/INS-GFP^+ in WT and KO-1 SC-β cells treated with high glucose for 24 h (two-way ANOVA with Sidak's multiple-comparisons test, $n = 3$). Data are presented as mean ± s.e.m. Individual data points are shown for all bar graphs. Source data are provided as a Source Data file.

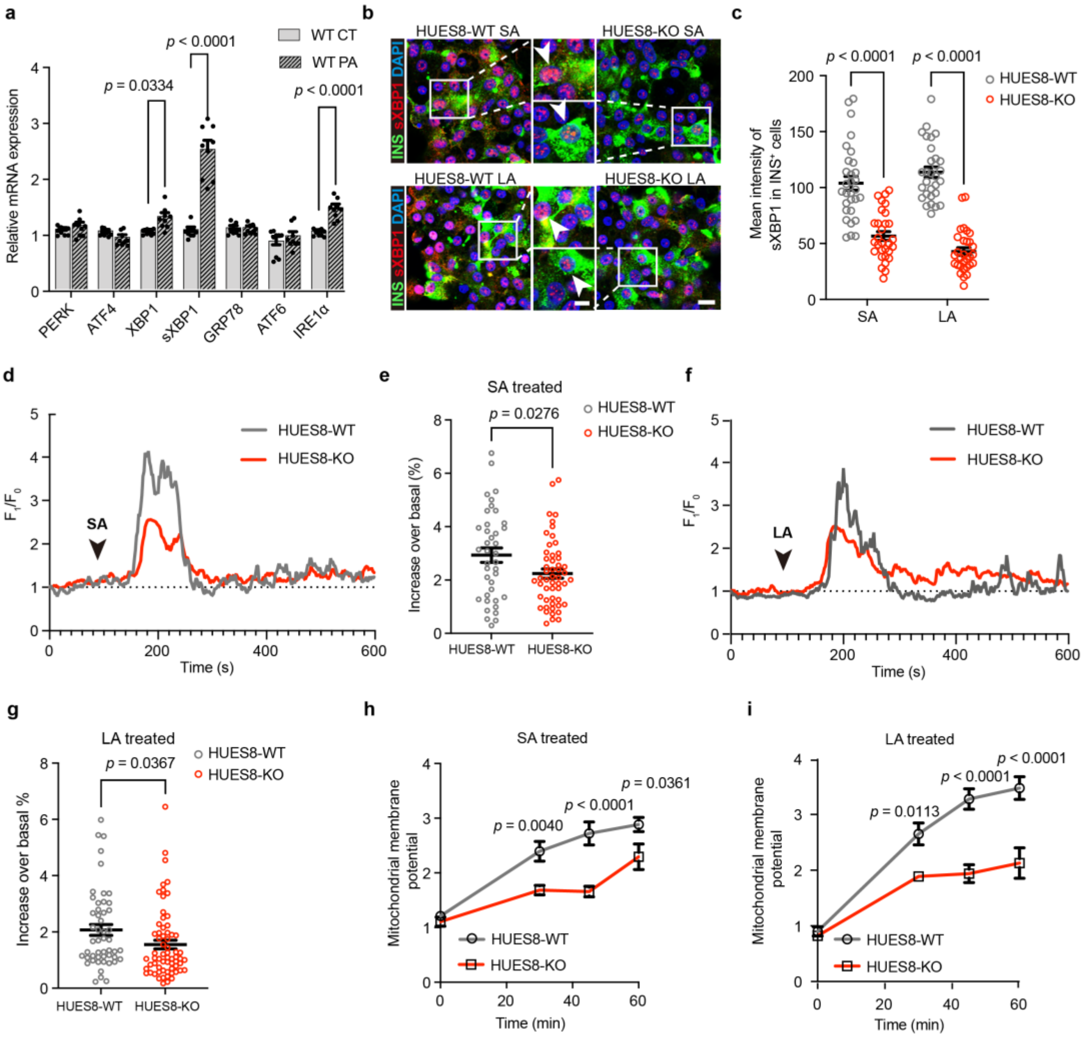

Supplementary Fig. 10, Additional analysis of ZnT8 LOF resistance to lipotoxicity-induced ER stress. a, qRT-PCR analysis of ER stress related genes expressed in WT SC-β cells in the absence or presence of PA (n = 9). **b**, **c**, Immunofluorescent images (**b**) and mean intensity measurements (**c**) of sXBP1 in the INS^{+} cells of HUES8-WT and KO SC-β cells treated with SA or LA for 24 h (n = 30 from 3 independent experiments). **d**, **f**, Representative trace of Fluo-4 relative fluorescence intensity (F_1/F_0) in a single SC-β cell of HUES8-WT and HUES8-KO upon SA (**d**) or LA (**f**) stimulation. **e**, **g**, Maximal peak increase in relative fluorescence intensity over average of baseline fluorescence intensity (0-90 s) upon SA (**e**; WT, n = 39; KO, n = 52 from 4 independent experiments) or LA (**g**; WT, n = 50; KO, n = 65. The data is from 4 independent experiments) stimulations. **h**, **i**, Mitochondrial membrane potential measured by JC-10 (mean fluorescence of monomer/aggregate). The samples were treated with SA (**h**; 0, n = 7; 30 min, n = 8; 45 min, n = 8; 60min, n = 7） or LA (**i**, HUES8-WT, n = 8; HUES8-KO, n = 7) for 0, 30 min, 45 min, and 60 min. Two-way ANOVA with Sidak's multiple-comparisons test was used to analyze for **a**, **c**, **h** and **i**; Unpaired two-tailed t-tests was used to analyze for **e** and **g**. Data are presented as mean ± s.e.m. Individual data points are shown for all bar graphs. Source data are provided as a Source Data file.

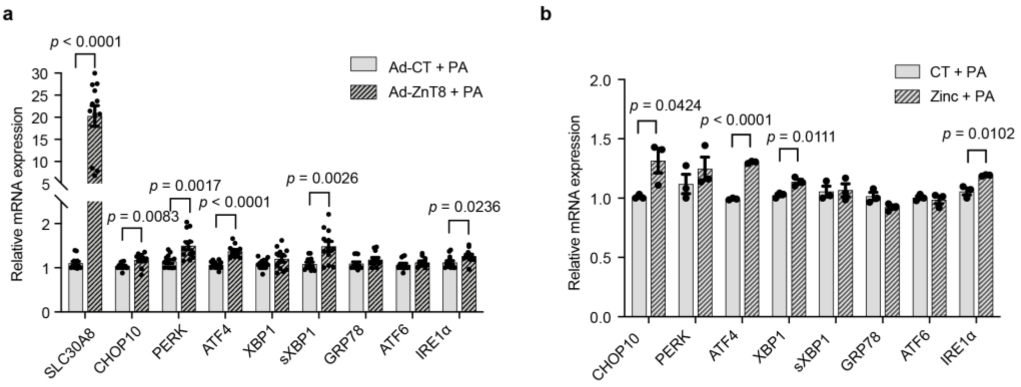

Supplementary Fig. 11, Excessive zinc induces ER stress in SC-β cells. a, qRT-PCR analysis of ER stress related genes in WT SC-β cells infected with adenovirus-ZnT8 or adenovirus-control infection in the presence of PA (*n* = 12). **b**, qRT-PCR analysis of ER stress related genes expressed in WT SC-β cells in the absence or in presence of extra zinc under PA treatment (*n* = 3). Unpaired two-tailed *t*-tests was used for analysis of **a** and **b**. Data are presented as mean ± s.e.m. Individual data points are shown for all bar graphs. Source data are provided as a Source Data file.

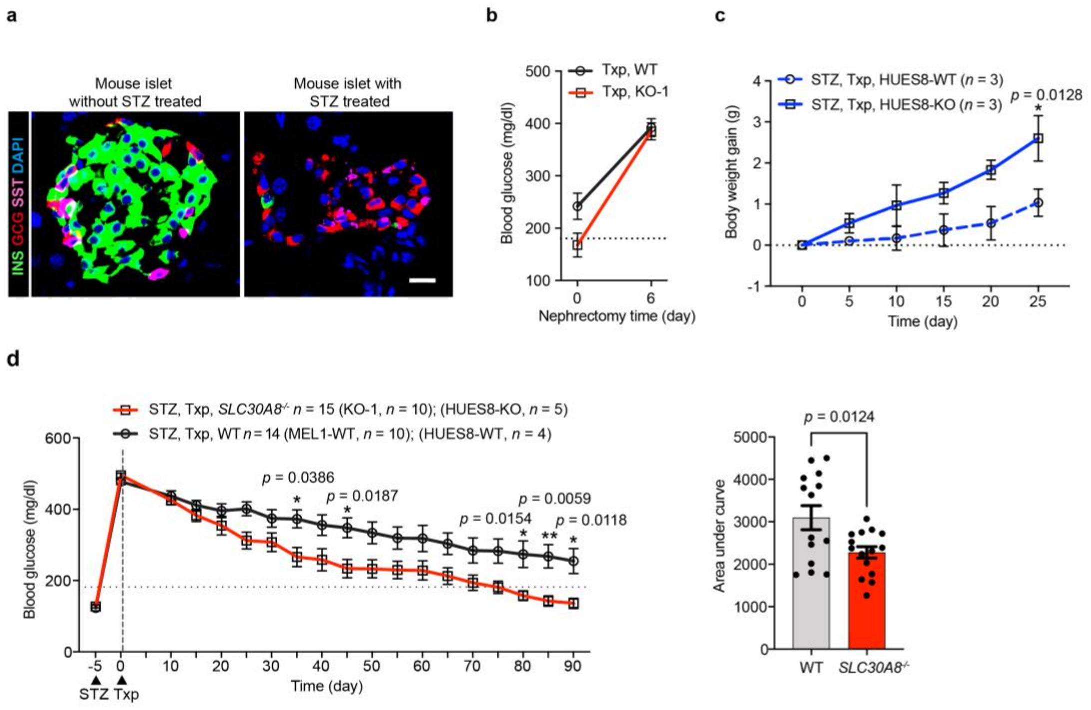

Supplementary Fig. 12, Additional analysis of the improvement of mice glycemic restoration with the transplant of ZnT8 LOF SC-β cell. a, Representative immunostaining images showing INS, GCG and SST in mouse islets with or without STZ treatment. Scale bar, 25 μm. **b**, Blood glucose level after nephrectomy (WT, *n* = 3; KO-1, *n* = 4) **c**, Body weight gain of the diabetic mice after transplantation (two-way ANOVA with Sidak's test for multiple comparisons, *n* = 3). **d**, Blood glucose levels (two-way ANOVA with Sidak's test for multiple comparisons) and AUC (unpaired two-tailed *t*-tests) of randomly fed STZ-induced diabetic mice transplanted with SC-β cells (black dotted line: STZ, WT Txp, *n* = 14; red line: STZ, KO Txp, *n* = 15). Data are presented as mean ± s.e.m. Individual data points are shown for all bar graphs. Source data are provided as a Source Data file.

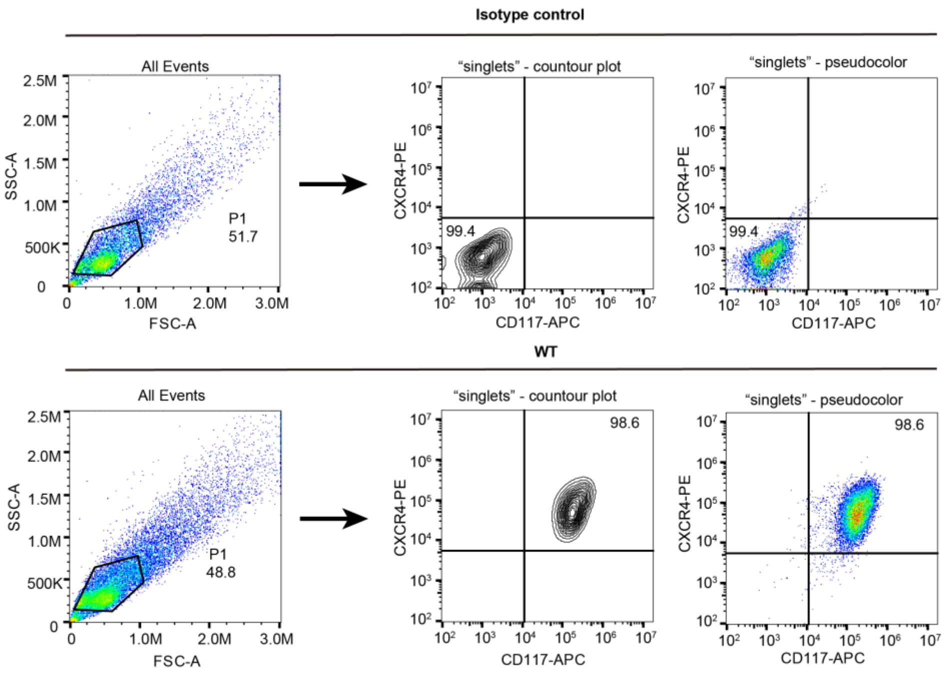

Supplementary Fig. 13, Flow cytometry gating strategy. The Flow Cytometry data was first gated on the basis of scatter properties and viability (P1). The subpopulations were gated based on the boundary of isotype-population and primary antibody-stained population to distinguish the positive and negative cells.

Supplementary Table 1, Information of cell lines.

**MEL1 *INS*$^{GFP/W}$**			
MEL1 *NKX6.1*$^{mCherry/mCherry}$-*INS*$^{GFP/W}$		mCherry insert	NKX6.1-mCherry
**MEL1 *NKX6.1*$^{mCherry/mCherry}$-*INS*$^{GFP/W}$**			
SLC30A8$^{-/-}$	KO-1	c. 479-524 del	p. Y160fs
	KO-2	c. 526-551 del	p. T176fs
**HUES8**			
SLC30A8$^{-/-}$	HUES8-KO	c. 525-550 del	p. T176fs
**MEL1 *NKX6.1*$^{mCherry/mCherry}$-*INS*$^{GFP/W}$**			
INS$^{-/-}$	INS KO	c.93-111 del	p. C31fs

Supplementary Table 2, sgRNA and sequencing primers of mutant cell lines

	sgRNA (5'-3')	Forward Primer Sequence	Reverse Primer Sequence
MEL1 *NKX6.1*$^{mCherry/mCherry}$-*INS*$^{GFP/W}$	GGCGTTCAGGATGAGCTCTC	CGACGAGAAAATCACGCAGCT	AACCTTGAAGTTGTGGGAGGGGAT
SLC30A8$^{-/-}$	GACTGGCGTGCTAGTGTACC	CCTTTTTGGGGGAAGTGGCAAAGT	CTAACCCTGCCTCTAGCACACCAGA
	CGATGATCATCACAGTCGCC		
INS$^{-/-}$	GGTGTGAGCCGCACAGGTGT	TGCGCCTCCTGCCCCTGCT	CCCAGCATGGGCAGAAGGGGGC

Supplementary Table 3, Primers used for real-time PCR

Gene Name	Forward Primer Sequence	Reverse Primer Sequence
SLC30A8	TTGCACCAGAGATGCCTTG	TCCAAGGGCATGCACAAA
INS	CAATGCCACGCTTCTGC	TTCTACACACCCAAGACCCG
CHOP10	TGACCAGGGAAGTAGAGGC	AGTGAGAGGGTAGTCAGTAGC
XBP1	CCTGGTTGCTGAAGAGGAGG	CCATGGGGAGATGTTCTGGAG
sXBP1	CTGAGTCCGCAGCAGGTG	TGCCCAACAGGATATCAGACT
ATF4	GACCGAAATGAGCTTCCTGA	ACCCATGAGGTTTGAAGTGC
PERK	GTCCGGAACCAGACGATGAG	GGCTGGATGACACCAAGGAA
IRE1α	CACAGTGACGCTTCCTGAAAC	GCCATCATTAGGATCTGGGAGA
GRP78	CACAGTGGTGCCTACCAAGA	TGATTGTCTTTTGTCAGGGGT
ATF6	TCCTCGGTCAGTGGACTCTTA	CTTGGGCTGAATTGAAGGTTTTG
PDX1	TGGAGCTGGCTGTCATGTTGA	CGCTTCTTGTCCTCCTCCTTTT
NKX6.1	CTGGCCTGTACCCCTCATCA	CTTCCCGTCTTTGTCCAACAA
MAFA	TTCAGCAAGGAGGAGGTCAT	CGCCAGCTTCTCGTATTTCT
SYT13	GGTCTGCACAACAGTTCAATG	CCACACCATTCTGAGGGAGG
VAMP2	CTCAAGCGCAAATACTGGTGG	TGATGGCGCAAATCACTCCC

Supplementary Table 4, Antibody list.

Antibody Target	Company	Catalog number	Dilution
Rabbit anti-OCT4	Abcam	ab19857	1:100
Mouse anti-NANOG	Cell Signaling Technology	4893S	1:100
CXCR4-PE	Invitrogen	MHCXCR404	1:100
CD117-APC	Invitrogen	CD11705	1:100
Goat anti-SOX17	R&D System	AF1924	1:100
Rabbit anti-FOXA2	EMD Millipore	07-633	1:100
Goat anti-PDX1	R&D Systems	AF2419	1:100
Mouse anti-NKX6.1	Developmental Studies Hybridoma Bank	F55A12-c	1:50
Rat anti-C-peptide	Developmental Studies Hybridoma Bank	GN-1D4	1:50
Guinea pig anti-Insulin	Dako	A0564	1:800
Rabbit anti-ZnT8	MyBioSource	MBS7050703	1:200
Rabbit anti-SYT13	Abcepta	AP5482a	1:100
Rabbit anti-Glucagon	Cell Signaling Technology	2760S	1:100
Goat anti-Somatostatin	Santa Cruz Biotechnology	sc-7819	1:100
Mouse anti-sXBP1	Biolegend	658802	1:600
Rabbit anti-IRE1α	Cell Signaling Technology	3294T	1:1000

Supplementary Table 5, Differentiation protocol

Day	Stage	basal medium	Factor	Final Concentration
Day 0	**Stage 1** (Definitive Endoderm)	RPMI1640	Activin A	100 ng/ml
			Chir99021	2 µM
Day 1-2			BMP4	0.25 ng/ml
			bFGF	5 ng/ml
			Activin A	100 ng/ml
			vEGF	10 ng/ml
			Ascorbic acid	50 µg/ml
Day 3-5	**Stage 2** (Primitive Gut Tube)	SFD	FGF10	50 ng/ml
			Wnt3a	3 ng/ml
			Ascorbic acid	50 µg/ml
Day 6-7	**Stage 3** (Posterior Foregut)	DMEM	Ascorbic acid	50 µg/ml
			SANT1	0.25 µM
			Retinoic acid	2 µM
			NOGGIN	50 ng/ml
			FGF10	50 ng/ml
Day 8-10	**Stage 4** (Pancreatic Progenitors)	DMEM	Ascorbic acid	50 µg/ml
			NOGGIN	50 ng/ml
			hEGF	50 ng/ml
			Nicotinamide	10 mM
Day 11-13	**Stage 5** (Endocrine Progenitors)	MCDB131	SANT1	0.25 µM
			Alk5i-II	10 µM
			LDN193189	100 nM
			Retinoic acid	0.05 µM
			hEGF	50 ng/ml
			Triiodothyronine	1 µM
Day 14-19	**Stage 6** (Immature SC-β Cell)	MCDB131	Alk5i-II	10 µM
			LDN193189	100 nM
			Gamma secretase inhibitor XX	100 nM
			hEGF	50 ng/ml
			Triiodothyronine	1 µM
Day 20-33	**Stage 7** (Mature SC-β Cell)	MCDB131	Triiodothyronine	1 µM
			Alk5i-II	10 µM
			Trolox	10 µM
			R428	2 µM
			N-Cys	1 mM
