# Supplementary material for: ZnT8 loss-of-function accelerates functional maturation of hESC-derived β cells and resists metabolic stress in diabetes
Source: Nat Commun. 2022 Jul 16;13:4142. doi: 10.1038/s41467-022-31829-9 (PMC9288460; doi:10.1038/s41467-022-31829-9)
Supplement: Supplementary file 2 — Reporting Summary [file 41467_2022_31829_MOESM2_ESM.pdf]

nature portfolio

Corresponding author(s): Weida Li

Last updated by author(s): May 29, 2022

Reporting Summary

Nature Portfolio wishes to improve the reproducibility of the work that we publish. This form provides structure for consistency and transparency in reporting. For further information on Nature Portfolio policies, see our Editorial Policies and the Editorial Policy Checklist.

Statistics

For all statistical analyses, confirm that the following items are present in the figure legend, table legend, main text, or Methods section.

n/a	Confirmed	
[ ]	[x]	The exact sample size (*n*) for each experimental group/condition, given as a discrete number and unit of measurement
[ ]	[x]	A statement on whether measurements were taken from distinct samples or whether the same sample was measured repeatedly
[ ]	[x]	The statistical test(s) used AND whether they are one- or two-sided<br>*Only common tests should be described solely by name; describe more complex techniques in the Methods section.*
[x]	[ ]	A description of all covariates tested
[ ]	[x]	A description of any assumptions or corrections, such as tests of normality and adjustment for multiple comparisons
[ ]	[x]	A full description of the statistical parameters including central tendency (e.g. means) or other basic estimates (e.g. regression coefficient) AND variation (e.g. standard deviation) or associated estimates of uncertainty (e.g. confidence intervals)
[ ]	[x]	For null hypothesis testing, the test statistic (e.g. *F*, *t*, *r*) with confidence intervals, effect sizes, degrees of freedom and *P* value noted<br>*Give P values as exact values whenever suitable.*
[x]	[ ]	For Bayesian analysis, information on the choice of priors and Markov chain Monte Carlo settings
[x]	[ ]	For hierarchical and complex designs, identification of the appropriate level for tests and full reporting of outcomes
[x]	[ ]	Estimates of effect sizes (e.g. Cohen's *d*, Pearson's *r*), indicating how they were calculated

Our web collection on statistics for biologists contains articles on many of the points above.

Software and code

Policy information about availability of computer code

Data collection	BD FACSVerse was used run samples, data were collected by BD FACSuite application.<br>Immunofluorescence images were captures by Leica SP8 confocal microscope.<br>The q-PCR data were acquired by using Roche LightCycler 96 instrument.<br>The submicroscopic structure of insulin granule was observed by transmission electron microscope of JEOL JEM-1230.<br>The absorbance value of ELISA was measured by Molecular Devices SpectraMax M5.
Data analysis	STAR (v2.6.0a) was used to do alignment.<br>Drop-seq (v1.13) was performed to quantify expression level of genes for each cell barcode.<br>Scater was conducted to quality control.<br>Seurat (v2.3.4) was used for downstream analysis: cell clustering and differential analysis between clusters.<br>ClusterProfiler (v3.10.0) was used for GO enrichment and GSEA.<br>Monocle (v2.0) was used for pseudotime analysis.<br>Custom scripts were available at https://github.com/wangmhan/pancreaticBetaKO<br>NCBI (https://www.ncbi.nlm.nih.gov) and SnapGene Viewer 6.0.2 were used for designing sgRNA and analyzing Sanger sequencing data.<br>Quantitative analysis was performed by GraphPad Prism 8.0. Flow cytometry analysis was performed by Flow Jo 10. Mean intensity or mean gray value of the images were analyzed by Image J 1.8.0.

For manuscripts utilizing custom algorithms or software that are central to the research but not yet described in published literature, software must be made available to editors and reviewers. We strongly encourage code deposition in a community repository (e.g. GitHub). See the Nature Portfolio guidelines for submitting code & software for further information.

Data

Policy information about availability of data

All manuscripts must include a data availability statement. This statement should provide the following information, where applicable:

- Accession codes, unique identifiers, or web links for publicly available datasets
- A description of any restrictions on data availability
- For clinical datasets or third party data, please ensure that the statement adheres to our policy

The single cell RNA-seq data of this study have been deposited in Gene Expression Omnibus (GEO) under accession code GSE135076 (https://www.ncbi.nlm.nih.gov/geo/query/acc.cgi?acc=GSE135076) and scripts for analyses can be found at https://github.com/wangmhan/pancreaticBetaKO. All relevant data are reported in the main text or supplementary information. The data supporting the findings of this study are available from the corresponding authors on request.

Field-specific reporting

Please select the one below that is the best fit for your research. If you are not sure, read the appropriate sections before making your selection.

[x] Life sciences   [ ] Behavioural & social sciences   [ ] Ecological, evolutionary & environmental sciences

For a reference copy of the document with all sections, see nature.com/documents/nr-reporting-summary-flat.pdf

Life sciences study design

All studies must disclose on these points even when the disclosure is negative.

Sample size	Statistical methods were not used to determine sample size on this study. Sample sizes were chosen based on availability of experimental samples. The sample sizes were sufficient since we used many experimental techniques to confirm the results.
Data exclusions	SC-β Cells differentiation that did not pass a minimum criteria of 93% CD117+/CXCR4+ double positive cells at the early stage of definitive endoderm were excluded from further analysis. This criteria was preselected because the formation of definitive endoderm is the first step toward pancreatic differentiation. If the cells don't pass the above criteria, the spheres will not form pancreatic endoderm efficiently and start falling apart on further culture. No data were excluded for other analyses of this study
Replication	All data could be reproduced, and all experiments and analyses presented were the result of at least 3 independent biological replicates. All attempts at replication were successful.
Randomization	For the in vivo data, animals were randomized using randomized number table to be transplanted with WT or KO SC-β cells. The in vitro data were descriptive the differences between WT and ZnT8 KO-derived SC-β cells, thus the randomization was not applicable.
Blinding	For the in vitro experiment, mean intensity measurement (Figure 1j, Figure 5d, i, Supplementary Figure 6c, Supplementary Figure 7a, c, g, i, Supplementary Figure 10c) and cell type quantifications (Figure. 6d) of images were blinded to the investigator to analyze. The in vivo experiments including Figure 2j-k, Figure 6b and 6d-g, Supplementary Figure 12b-d were blinded to data collectors. Other cell biology essays were not blinded to the collectors and investigators according to our standard laboratory practice.

Reporting for specific materials, systems and methods

We require information from authors about some types of materials, experimental systems and methods used in many studies. Here, indicate whether each material, system or method listed is relevant to your study. If you are not sure if a list item applies to your research, read the appropriate section before selecting a response.

Materials & experimental systems

n/a	Involved in the study	
[ ]	[x]	Antibodies
[ ]	[x]	Eukaryotic cell lines
[x]	[ ]	Palaeontology and archaeology
[ ]	[x]	Animals and other organisms
[x]	[ ]	Human research participants
[x]	[ ]	Clinical data
[x]	[ ]	Dual use research of concern

Methods

n/a	Involved in the study	
[x]	[ ]	ChIP-seq
[ ]	[x]	Flow cytometry
[x]	[ ]	MRI-based neuroimaging

Antibodies

Antibodies used	Rabbit Anti-OCT4, 1:100; Applicable: ICC/IF; Provide supplier name: Abcam; Catalog number: ab19857; Clone name: unknown; Lot

	number: GP315681-7<br>Mouse Anti-NANOG, 1:100; Applicable: ICC/IF; Provide supplier name: Cell Signaling Technology; Catalog number: 4893S; Clone name: 1E6C4; Lot number: 5<br>CXCR4-PE, 1:100; Applicable:FACS; Provide supplier name: Invitrogen; Catalog number:MHCXCR404; Clone number: 12G5<br>CD117-APC, 1:100; Applicable:FACS; Provide supplier name: Invitrogen; Catalog number:CD11705; Clone number: 104D2<br>Goat Anti-SOX17, 1:100; Applicable: IF; Provide supplier name: R&D Systems; Catalog number: AF1924; Clone name: unknown; Lot number: KGA0916091<br>Rabbit Anti-FOXA2, 1:100; Applicable: IF; Provide supplier name: MilliporeSigma; Catalog number: 07-633; Clone name: unknown; Lot number: 2768454<br>Goat Anti-PDX1, 1:100; Applicable: IF; Provide supplier name: R&D Systems; Catalog number: AF2419; Clone name: unknown; Lot number: UNY0117051<br>Mouse Anti-NKX6.1, 1:50; Applicable: IF/FACS; Provide supplier name: Developmental Studies Hybridoma Bank; Catalog number: F55A12-c; Clone name: unknown; Lot number: unknown<br>Rat Anti-C-peptide, 1:50; Applicable: IF/FACS; Provide supplier name: Developmental Studies Hybridoma Bank; Catalog number: GN-1D4; Clone name: unknown; Lot number: unknown<br>guinea pig Anti-Insulin, 1:800; Applicable: IF; Provide supplier name: Dako; Catalog number: A0564; Clone name: unknown; Lot number: 10112678<br>Rabbit Anti-ZnT8, 1:200; Applicable: IF; Provide supplier name: MyBioSource; Catalog number: MBS7050703; Clone name: unknown; Lot number: F0811A<br>Rabbit Anti-Glucagon, 1:100; Applicable: IF; Provide supplier name: Cell Signaling Technology; Catalog number: 2760S; Clone name: unknown; Lot number: 2<br>Goat Anti-Somatostatin, 1:100; Applicable: IF; Provide supplier name: Santa Cruz; Catalog number: sc-7819; Clone name: G-10; Lot number: E2716<br>Mouse Anti-sXBP1, 1:600; Applicable: IF; Provide supplier name: Biolegend; Catalog number: 658802; Clone name: unknown; Lot number: 9D11A43<br>Rabbit Anti-IRE1α, 1:1000; Applicable: WB; Provide supplier name: Cell Signaling Technology; Catalog number: 3294T; Clone name: 14C10; Lot number: 5<br>Rabbit Anti-SYT13, 1:100;Applicable: IF; Provide supplier name: Abcepta;Catalog number: AP5482a;Clone name: unknown
Validation	Anti-OCT4, Species reactivity: human; Application: IF; Relevant citations: PMID:32710237; PMID:32757137<br>Anti- NANOG, Species reactivity: human; Application: IF; Relevant citations: PMID: 18358816; PMID: 18029452<br>CXCR4-PE, Species reactivity: human; Application: FACS; Relevant citations: PMID: 27148368<br>CD117-APC, Species reactivity: human; Application: IFACS; Relevant citations: PMID:30970261<br>Anti- SOX17, Species reactivity: human; Application: IF; Relevant citations: PMID: 11973269; PMID: 11786926<br>Anti-FOXA2, Species reactivity: human; Application: IF; Relevant citations: PMID: 25889591; PMID: 24371808<br>Anti-PDX1, Species reactivity: human; Application: IF; Relevant citations: PMID: 31493350; PMID: 31291575<br>Anti-NKX6.1, Species reactivity: human; Application: IF; Relevant citations: PMID: 25402613; PMID: 29326366<br>Anti-C-peptide, Species reactivity: human; Application: IF; Relevant citations: PMID: 23468018; PMID: 26216140<br>Anti-Insulin, Species reactivity: human; Application: IF; Relevant citations: PMID: 31509750; PMID: 22980982<br>Anti-Glucagon, Species reactivity: human; Application: IF; Relevant citations: PMID: 27935966; PMID: 25751815<br>Anti- Somatostatin, Species reactivity: human; Application: IF; Relevant citations: PMID: 26021489; PMID: 24252877<br>Anti-ZnT8, Species reactivity: human; Application: IF; Relevant citation: PMID: 26824044<br>Anti-sXBP1, Species reactivity: human; Application: IF; Relevant citations: PMID: 28504640; PMID: 28921568<br>Anti- IRE1α, Species reactivity: human; Application: WB; Relevant citations: PMID: 12042763; PMID: 1625574<br>Anti-SYT13,Species reactivity: human; Application: IF; Relevant citation: PMID:32029226

Eukaryotic cell lines

Policy information about cell lines

Cell line source(s)	MEL1-INS GFP/W hESCs were provided by Drs. E. Stanley and Dr. A. Elefanty;MEL1 NKX6.1mCherry/mCherry-INSGFP/W hESCs gene-edited cell line was generated by the co-author Yini Xiao; SLC30A8 Knockout cell lines were generated by the co-author Qing Ma;INS Knockout cell line was generated by the co-author Rui Hu;HUES8 hESCs were provided by Dr.Qiurong Ding.
Authentication	We designed the primers and used PCR to authenticate all of the gene-edited cell lines. And then we compared the PCR products with the original DNA sequence to determine whether the target fragments have been successfully edited. All the cell lines were under authentication.
Mycoplasma contamination	All cell lines were checked to be mycoplasma-free once a month by PCR assay.
Commonly misidentified lines<br>(See ICLAC register)	No misidentified cell line was used in this study.

Animals and other organisms

Policy information about studies involving animals; ARRIVE guidelines recommended for reporting animal research

Laboratory animals	Species: mouse; Strain:SCID-Beige; Sex:male; Age:6-8 weeks.The mice were housed in a constant temperature room (22 ± 1 °C) with a 12-h light/dark cycle and 40–60% humidity.

Wild animals	The study did not involve wild animals.
Field-collected samples	The study did not involve samples collected from field.
Ethics oversight	All experiments were performed in accordance with the University of Health Guide for the Care and Use of Laboratory Animals and were approved by the Biological Research Ethics Committee of Tongji University.

Note that full information on the approval of the study protocol must also be provided in the manuscript.

Flow Cytometry

Plots

Confirm that:

- [x] The axis labels state the marker and fluorochrome used (e.g. CD4-FITC).
- [x] The axis scales are clearly visible. Include numbers along axes only for bottom left plot of group (a 'group' is an analysis of identical markers).
- [x] All plots are contour plots with outliers or pseudocolor plots.
- [x] A numerical value for number of cells or percentage (with statistics) is provided.

Methodology

Sample preparation	Approximately 10^6 cells were dissociated into single cells with 0.25% trypsin and fixed with 1.6% PFA in 37°C for 20 min. The cells were washed with dPBS and permeabilized by 10% Saponin buffer (Biolegend, #421002) for 5 min. The permeabilized cells were incubated in primary antibodies for 30 min at RT. After washed with FACS buffer for 2 times, cells were incubated with fluorescence-conjugated secondary antibodies for 30 min at RT.
Instrument	BD FACSVerse; CytoFLEX LX; Moflo Astrios 4 lasers.
Software	Flow Jo 10.
Cell population abundance	No cell sorting was conducted.
Gating strategy	The Flow Cytometry data Was first gated on the basis of scatter properties and viability. The subpopulations were gated based on the boundary of isotype-population and primary antibody-stained population to distinguish double-positive and negative cells.

- [x] Tick this box to confirm that a figure exemplifying the gating strategy is provided in the Supplementary Information.
